# Large‐Scale Quantitative Morphometry of Platelet α‐Granules via SIM Super‐Resolution Microscopy for Cancer Liquid Biopsy

**DOI:** 10.1002/advs.75094

**Published:** 2026-04-10

**Authors:** Yan Ma, Huan Deng, Zelin Liu, Simei Zhong, Liangliang Wang, Mu He, Ning Jing, Lihua Dai, Yuzhen Basang, Xiao‐Yu Hu, Changyi Zhang, Huimin Zeng, Haoren Shao, Zhe Yang, Shanshan Zhao, Xiaohai Hu, Chuanjie Zhang, Xiaowei Wu, Jing Xu, Shaoqun Zeng, Jing Yuan, Qiang Li, Ziwei Qu, Zhenya Hong, Lingjuan Chen, Hanhua Dong, Jia Guo, Zhiqiang Han, Yu‐Hui Zhang

**Affiliations:** ^1^ MOE Key Laboratory for Biomedical Photonics Advanced Biomedical Imaging Facility‐Wuhan National Laboratory for Optoelectronics Huazhong University of Science and Technology Wuhan China; ^2^ Institute of Blood Transfusion of Hubei Province Wuhan Blood Center Wuhan China; ^3^ Department of Obstetrics and Gynecology Tongji Hospital Tongji Medical College Huazhong University of Science and Technology Wuhan China; ^4^ Department of Urology Renmin Hospital of Wuhan University Wuhan China; ^5^ Hepatic Surgery Center Tongji Hospital Tongji Medical College Huazhong University of Science and Technology Wuhan China; ^6^ Department of Thoracic Surgery Tongji Hospital Tongji Medical College Huazhong University of Science and Technology Wuhan China; ^7^ Department of Gastrointestinal Surgery Traditional Chinese and Western Medicine Hospital of Wuhan Tongji Medical College Huazhong University of Science and Technology Wuhan China; ^8^ Department of Hematology Tongji Hospital Tongji Medical College Huazhong University of Science and Technology Wuhan China; ^9^ Cancer Center Union Hospital Tongji Medical College Huazhong University of Science and Technology Wuhan China; ^10^ Hubei Key Laboratory of Precision Radiation Oncology Wuhan China; ^11^ Department of Hepatobiliary Surgery Shanxi Bethune Hospital, Shanxi Academy of Medical Sciences Tongji Shanxi Hospital Third Hospital of Shanxi Medical University Taiyuan China; ^12^ Department of Obstetrics and Gynecology Shanxi Bethune Hospital, Shanxi Academy of Medical Sciences Tongji Shanxi Hospital Third Hospital of Shanxi Medical University Taiyuan China

## Abstract

Blood‐based liquid biopsies hold transformative potential for non‐invasive cancer management, but current approaches relying on rare circulating tumor components limit their broad clinical utility. Platelets, abundant in blood and mediating diverse cancer‐associated responses, represent a compelling yet largely unexplored alternative. Using SIM super‐resolution microscopy, we analyzed α‐granule distributions in platelets from a multicenter cohort (*n* = 1,556) encompassing nine cancer types and twelve non‐malignant diseases. We identified robust cancer‐associated alterations, particularly a marked increase in the “Circle” pattern, which exhibits excellent multi‐cancer diagnostic potential. This method, termed PAID (Platelet Alpha‐granule Imaging‐based Diagnostic assay), achieved 85.0% accuracy in prostate cancer within the diagnostically challenging PSA “gray zone”. PAID combined with PSA improved diagnostic accuracy to 94.2%, 38.4% over PSA alone, suggesting significant synergy. In ovarian cancer, PAID combined with HE4 enhanced diagnostic accuracy from 73.8% to 88.3%. Furthermore, PAID outperformed CA125 in sensitivity (90.3% vs 60.0%) for detecting ovarian cancer recurrence. We also observed that tumor cells may utilize both tumor‐derived exosomes and proteins to remodel platelet α‐granule distributions, suggesting the increased “Circle” pattern arises from synergistic rather than singular triggers. The non‐invasive, highly accurate PAID shows great promise in advancing liquid biopsy for cancer management.

## 1 Introduction

Blood‐based liquid biopsies hold immense promises to revolutionize cancer management by offering non‐invasive routes to screening, diagnosis, prognosis, and therapy monitoring [1, 2]. Despite the clinical allure of analyzing circulating tumor components such as DNA and cells, current liquid biopsy approaches face critical limitations [3, 4]. The scarcity of tumor‐derived components in blood, which are often overshadowed by normal components [5, 6, 7], and a lack of standardized workflows, which leads to inconsistent results, hamper the widespread clinical utility and reliability [8, 9]. Thus, the field urgently requires novel blood‐based tools to unlock the full potential of liquid biopsies in cancer care. Platelets, essential mediators of both local and systemic responses in cancer, represent a compelling yet largely unexplored alternative for liquid biopsies [10, 11, 12]. Unlike rare circulating tumor cells or fragmented DNA, platelets are readily abundant in blood and easily isolated, presenting inherent practical advantages [12, 13, 14].

Recent studies leveraging electron or super‐resolution microscopy to overcome the imaging limitations posed by the ultra‐small size of platelet subcellular structures have uncovered a new dimension: cancer‐associated subcellular morphological changes within platelets themselves [15, 16]. These alterations, especially in organelles like microtubules, mitochondria, and α‐granules, represent a rich, untapped source of disease‐relevant information [15, 16]. Our prior work [15] has identified distinct alterations in platelet α‐granules distribution patterns in patients with cervical, endometrial, and ovarian cancers. This ultrastructural remodeling within platelets indicates a potential novel tumor‐indicating signal independent of traditional molecular markers, thereby necessitating a re‐evaluation of platelet involvement in tumor diagnosis and potentially opening avenues for new non‐invasive detection strategies. Nevertheless, whether these specific α‐granule alterations represent a robust biological pattern generalizable across diverse cancer types and whether their characteristic features possess sufficient diagnostic accuracy to serve as reliable diagnostic marker remain undetermined. This uncertainty constitutes a critical gap in tumor biology and a central obstacle to translating these preliminary observations into broadly applicable diagnostic tools. Consequently, extensive and rigorous validation of their potential as non‐invasive biomarkers would offer transformative solutions for oncology.

To address these critical gaps, we conducted a large‐scale analysis of platelet α‐granule distributions using structured illumination super‐resolution microscopy (SIM) in a cohort of 1,556 individuals spanning nine cancer types, twelve non‐malignant diseases, and five medical centers. We identified robust cancer‐associated alterations in α‐granule distributions, particularly a notable increase in the proportion of the “Circle” pattern, which exhibits excellent discriminatory capability for multi‐cancer detection compared with inflammatory or benign controls. We designated this method—leveraging the “Circle” pattern identified via super‐resolution imaging—PAID (Platelet Alpha‐granule Imaging‐based Diagnostic assay). Notably, combining PAID with existing clinical biomarkers like PSA (prostate‐specific antigen) and HE4 (human epididymis protein 4) remarkably enhances the diagnostic accuracy for prostate cancer (from 55.8% to 94.2%) and ovarian cancer (from 73.8% to 88.3%), compared with using PSA or HE4 alone. Additionally, PAID surpassed CA125 (cancer antigen 125) in sensitivity for detecting ovarian cancer recurrence (90.3% vs 60.0%). Furthermore, we found that tumor‐secreted exosomes and proteins cooperatively drive the increase in the “Circle” pattern, suggesting that this phenotype arises from the concerted action of multiple factors rather than a single trigger. This work not only establishes a non‐invasive, highly accurate diagnostic strategy with significant translational potential, but also considerably broadens the scope of liquid biopsy for cancer diagnosis.

## 2 Results

### 2.1 Platelet α‐granule Morphology: A Novel Signature for Cancer Diagnosis

Platelet α‐granules, critical regulators of diverse physiological and pathological processes [17], may undergo cancer‐associated alterations. However, their nanoscale size (150–300 nm) impedes detailed analysis by conventional optical microscopy [18]. Electron microscopy provides sufficient resolution but suffers from low throughput, restricting its clinical applicability [19]. The emergence of super‐resolution microscopy, particularly SIM, which combines high imaging speed with adequate spatial resolution, has overcome these limitations [18, 19]. Using SIM, we previously established, for the first time, a high‐throughput super‐resolution imaging workflow for platelet subcellular structures. This workflow enables analysis of platelet subcellular structures down to tens of nanometers, identifying α‐granule distributions as a promising target for cancer detection. However, these initial findings were observed in small cohorts (144 patients from five cancer types) [15], thus raising critical questions about their robustness—whether they represent true biological phenomena or are merely artifacts resulting from limited patient observation, and whether they possess sufficient diagnostic accuracy to serve as reliable diagnostic markers.

**FIGURE 1:**
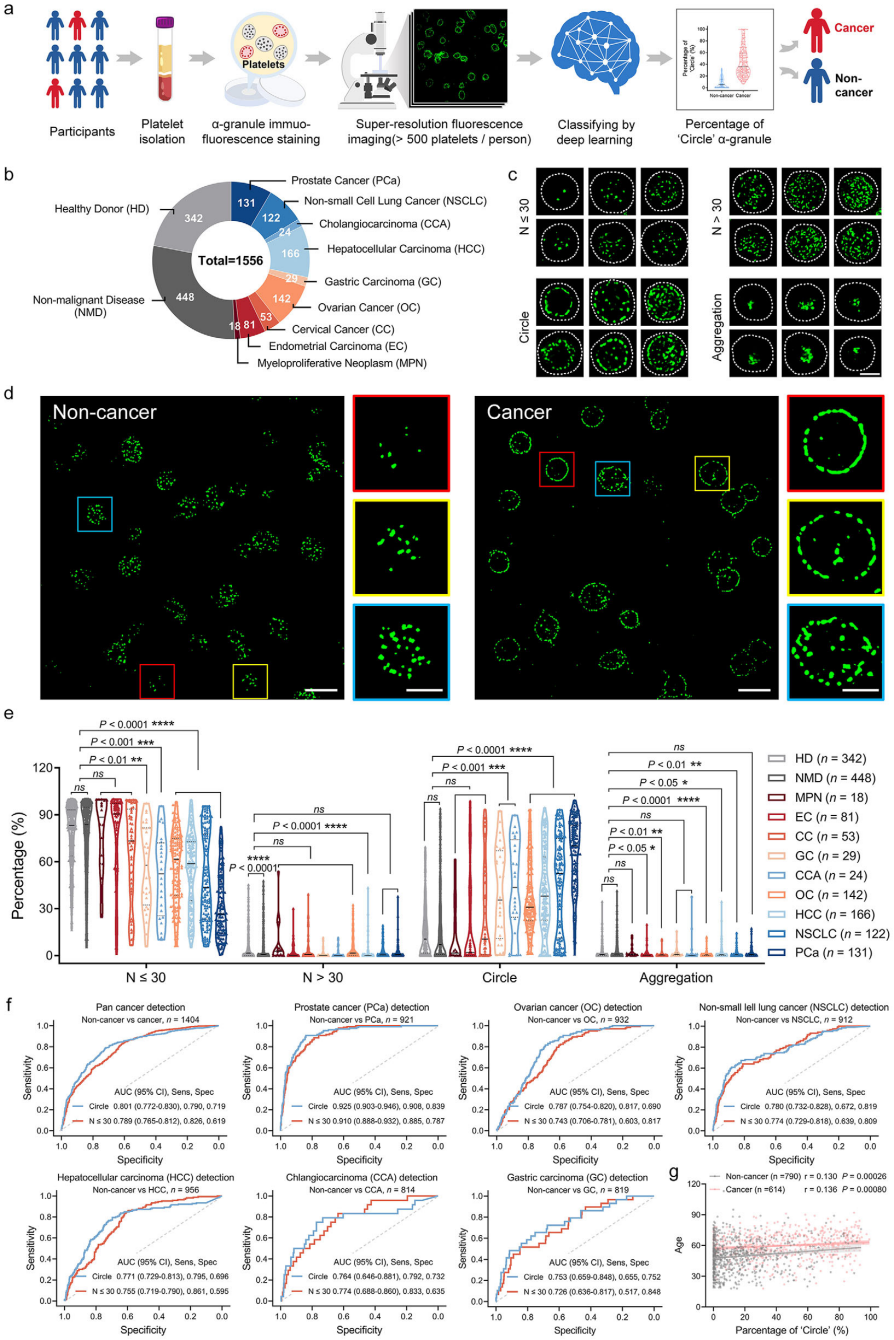
Platelet α‐granule distribution patterns differentiate cancers. a, Workflow schematic for unbiased analysis using standardized sample processing and custom deep learning image classification. Ethical approval: see Methods. b, Distribution of participants (*n* = 1,556). Study cohort included HD, NMD, and patients with nine cancers. Demographic details are in Extended Data Table S1. c, Representative SIM images of α‐granule distribution patterns: N ≤ 30 (a scattered dot pattern with 30 or fewer dots in each platelet), N > 30 (a scattered dot pattern with more than 30 dots in each platelet), Circle (a circle of dots distributed around the cell membrane), and Aggregation (aggregation in a small region). The white dotted line represents the boundary of a platelet. Scale bar, 2 µm. d, Representative SIM images of α‐granule distributions in non‐cancer controls (left, “N ≤ 30” pattern, including HD and NMD) and cancer patients (right, “Circle” pattern). Zoomed insets show boxed regions. Scale bar, 5 µm (main); 2 µm (insets). e, Percentages of α‐granule distribution patterns in non‐cancer controls and cancer patients (violin plots; median (solid line), interquartile range (IQR) (dashed lines)). The percentage of “Circle” pattern was significantly increased, and the percentage of “N ≤ 30” pattern was decreased in cancer patients (Kruskal–Wallis test with Bonferroni correction; *P* values in Extended Data Table S3). f, ROC curves evaluate the diagnose performance of “Circle” and “N ≤ 30” patterns for distinguishing cancers from non‐cancer controls (*n* = 790). AUC (95% CI), sensitivity (Sens), and specificity (Spec) are indicated. “Circle” pattern achieved an AUC of 0.801 for all cancers combined, excluding MPN, CC, and EC, with individual cancer types exhibiting good discrimination (AUC > 0.75), and demonstrated the highest accuracy in PCa showed highest accuracy (AUC = 0.925). g, Correlation between age and “Circle” pattern in cancer patients and non‐cancer controls. Spearman's rank correlation coefficient (r) indicates no significant correlation.

To rigorously evaluate the clinical relevance of platelet α‐granule distribution alterations, we conducted a multi‐center study encompassing a significantly enlarged cohort of 1,556 individuals. Inclusion criteria of this research include: (1) healthy donors confirmed by clinical examination and laboratory tests to have no malignancies, severe systemic diseases, or hematological abnormalities; patients with histologically or clinically confirmed benign, non‐malignant diseases; patients with cancer pathologically confirmed by surgical or biopsy specimens; (2) participants who had not received surgery, chemotherapy, radiotherapy, or immunotherapy before blood sampling; and (3) participants who had not taken aspirin, clopidogrel, or other antiplatelet/anticoagulant drugs within two weeks prior to blood collection. Exclusion criteria are: (1) individuals with a history or presence of diabetes mellitus and hematological disorders; (2) individuals who had received corticosteroids, immunosuppressive agents, or cytotoxic drugs within two weeks before blood collection; and (3) individuals who experienced acute thrombotic or major hemorrhagic events within three months prior to enrollment. Our high‐throughput workflow combines whole blood collection, platelet isolation, immunofluorescence staining, and SIM imaging, followed by automated deep‐learning segmentation and classification (Figure 1a). The large‐scale analysis included 766 patients across nine prevalent malignancies (Figure 1b). A carefully matched control group (*n* = 790) was established, comprising 344 healthy donors (HD) and 446 patients with 12 distinct non‐malignant diseases (NMD), encompassing ovarian cyst, fallopian tube cyst, chronic salpingitis, uterine myoma, endometrial polyps, endometrial hyperplasia, uterine prolapse, cervical intraepithelial neoplasia grade III, benign pulmonary nodule, benign prostatic hyperplasia, cirrhosis of the liver, and hepatic hemangioma (Extended Data Table S1). This robust, multi‐disease design provides the necessary statistical power and comparative framework to dissect the diagnostic significance of cancer‐associated platelet α‐granule distribution alternations. The study was performed with ethical approval from participating hospitals (see Methods).

As illustrated in Figure 1c, α‐granule distributions were categorized into four distinct patterns: N ≤ 30, N > 30, Circle, and Aggregation. In non‐cancer individuals (including HD and NMD), α‐granule predominantly displayed an “N ≤ 30” pattern (83.2%; Figure 1d, Extended Data Figure S1 and Extended Data Figure S2). Solid cancers, including gastric cancer (GC), cholangiocarcinoma (CCA), ovarian cancer (OC), hepatocellular carcinoma (HCC), non‐small cell lung cancer (NSCLC), and prostate cancer (PCa), exhibited a striking shift characterized by increased percentages of “Circle” and decreased percentages of “N ≤ 30” patterns (Figure 1d,e, Extended Data Figure S1 and Extended Data Figure S2). Previous studies have shown that platelet activation triggers α‐granule redistribution, typically centripetal centralization along with filopodia formation [20, 21]. The higher percentages of the “Circle” pattern observed in cancer‐patient platelets likely represent a cancer‐associated phenotype rather than classical activation, as it is spatially distinct from the canonical centripetal redistribution and lacks prominent filopodia. To corroborate this distinction, we stimulated platelets from healthy donors and cancer patients with thrombin and monitored α‐granule spatial organization over time (0, 2, 5, 10, and 15 min). In the absence of thrombin stimulation, platelets from both groups retained a smooth, discoid morphology (Extended Data Figure S3). Upon thrombin stimulation, platelets formed filopodia and redistributed α‐granules centripetally to the cell center, in agreement with previous reports [20, 21] (Extended Data Figure S3). Notably, cancer‐patient platelets exhibiting the “Circle” pattern transitioned from a peripheral ring to a central granular distribution upon the activation. Thus, the peripheral, ring‐like “Circle” pattern is distinct from the activation‐associated redistribution and is therefore unlikely to represent an in vitro artifact resulting from activation propensity. Moreover, although live‐cell labelling of α‐granules is not yet feasible owing to the lack of a live‐cell α‐granule probe, our previous work [15] using a live‐cell microtubule probe showed that the marginal‐band microtubule ring, an ultrastructural feature highly sensitive to activation, remains intact and comparable in live resting platelets and in fixed platelets processed with our protocol, indicating that the sample preparation procedure itself does not induce in vitro activation. We further assessed α‐granule morphology in platelets from whole blood stored at room temperature for 0, 12 and 24 h. The structural features remained stable over 24 h (Extended Data Figure S4). Collectively, these results indicate that the “Circle” pattern is unlikely to be an activation artifact induced by sample processing.

Remarkably, the percentages of “Circle” pattern were significantly enriched 4‐ to 9‐fold in OC, HCC, NSCLC, and PCa (median: 31%, 38.1%, 52.4%, and 70.1%, respectively) compared to non‐cancer individuals (median: 8.4%; *P* < 0.0001; Extended Data Table S2 and S3). “N ≤ 30” pattern also showed significant differences between these cancers and non‐cancer controls (*P* < 0.0001; Figure 1e and Extended Data Table S3), exhibiting similar discriminatory power. In contrast, myeloproliferative neoplasms (MPN), cervical cancer (CC), and endometrial cancer (EC) showed largely unchanged percentages of “N ≤ 30” and “Circle” patterns (Figure 1e and Extended Data Figure S1). Moreover, no obvious difference was observed between healthy individuals and patients with cirrhosis (median: 10.6 % vs 8.1 %, *P* = 0.873, Extended Data Figure S5a). Given that cirrhosis is widely recognized as a prototypical state of systemic inflammation, our result indicates that inflammation alone does not increase the abundance of this “Circle” pattern. In contrast, patients with HCC exhibited a significantly higher “Circle” proportion than those with cirrhosis (median: 38.1 % vs 8.1 %, P < 0.001, Extended Data Figure S5a). Furthermore, HCC patients with or without underlying cirrhosis displayed statistically indistinguishable “Circle” proportions (median: 35.9 % vs 39.7 %, *P* = 0.865, Extended Data Figure S5b). These findings reveal α‐granule distribution alterations uniquely associated with a subset of cancers, not observed in inflammatory and benign conditions. The percentages of “Circle” and “N ≤ 30” patterns, due to their discriminatory power, are identified as promising biomarkers for non‐invasive cancer detection.

Then, we explored the diagnostic potential of “Circle” and “N ≤ 30” patterns as liquid biopsy biomarkers. Receiver operating characteristic (ROC) curve analysis revealed that “Circle” pattern exhibits significant diagnostic performance, achieving an overall area under the curve (AUC) of 0.801 (95% confidence interval (CI) 0.772‐0.830, *n* = 1,404, excluding MPN, CC and EC) in differentiating cancer patients from non‐cancer individuals (Figure 1f). Notably, “Circle” pattern consistently demonstrated robust diagnose performance across six diverse cancer types, with AUC values exceeding 0.75 in each case (Figure 1f and Extended Data Table S4), suggesting its broad potential clinical applicability for cancer diagnosis. Particularly noteworthy was its exceptional discriminatory power in prostate cancer (PCa), where it yielded an AUC of 0.925 (95% CI 0.903‐0.946), with high sensitivity (90.8%) and specificity (83.9%, Figure 1f and Extended Data Table S4). “N ≤ 30” pattern also showed promising discriminatory power; however, its AUC values were generally lower than those of “Circle” pattern (Figure 1f and Extended Data Table S4). Therefore, in subsequent experiments, only “Circle” pattern was utilized for further diagnostic applications. Moreover, the percentage of “Circle” pattern was found to be independent of age, as evidenced by the absence of a significant correlation in both cancer patients and non‐cancer individuals (Spearman's rank correlation coefficient (*r*) = 0.136 and 0.130, respectively; Figure 1g). Except for NSCLC and PCa, no significant stage‐dependent variation in “Circle” pattern proportions was detected across the other six cancer types examined (Extended Data Figure S6). Nevertheless, the marked imbalance in sample sizes across stages within individual cancer types limits definitive conclusions, warranting further investigation. These findings highlight the significant potential of platelet α‐granule morphology, particularly “Circle” pattern, as a readily accessible diagnostic signature. Consequently, our PAID leveraging “Circle” pattern holds significant promise as a novel diagnostic strategy for cancer.

To demonstrate the clinical utility of PAID, we subsequently focused on PCa and OC among the four cancer types with the most pronounced platelet alterations (OC, HCC, NSCLC, and PCa) because the “Circle” pattern achieved the highest diagnostic performance in these two diseases (AUC = 0.925 for PCa and 0.787 for OC; Figure 1f). Moreover, both cohorts contained roughly equal numbers of malignant and benign samples (approximately 1:1), permitting unbiased assessment.

### 2.2 PAID Outperforms Clinical PSA in Prostate Cancer (PCa) Screening and Diagnosis

**FIGURE 2:**
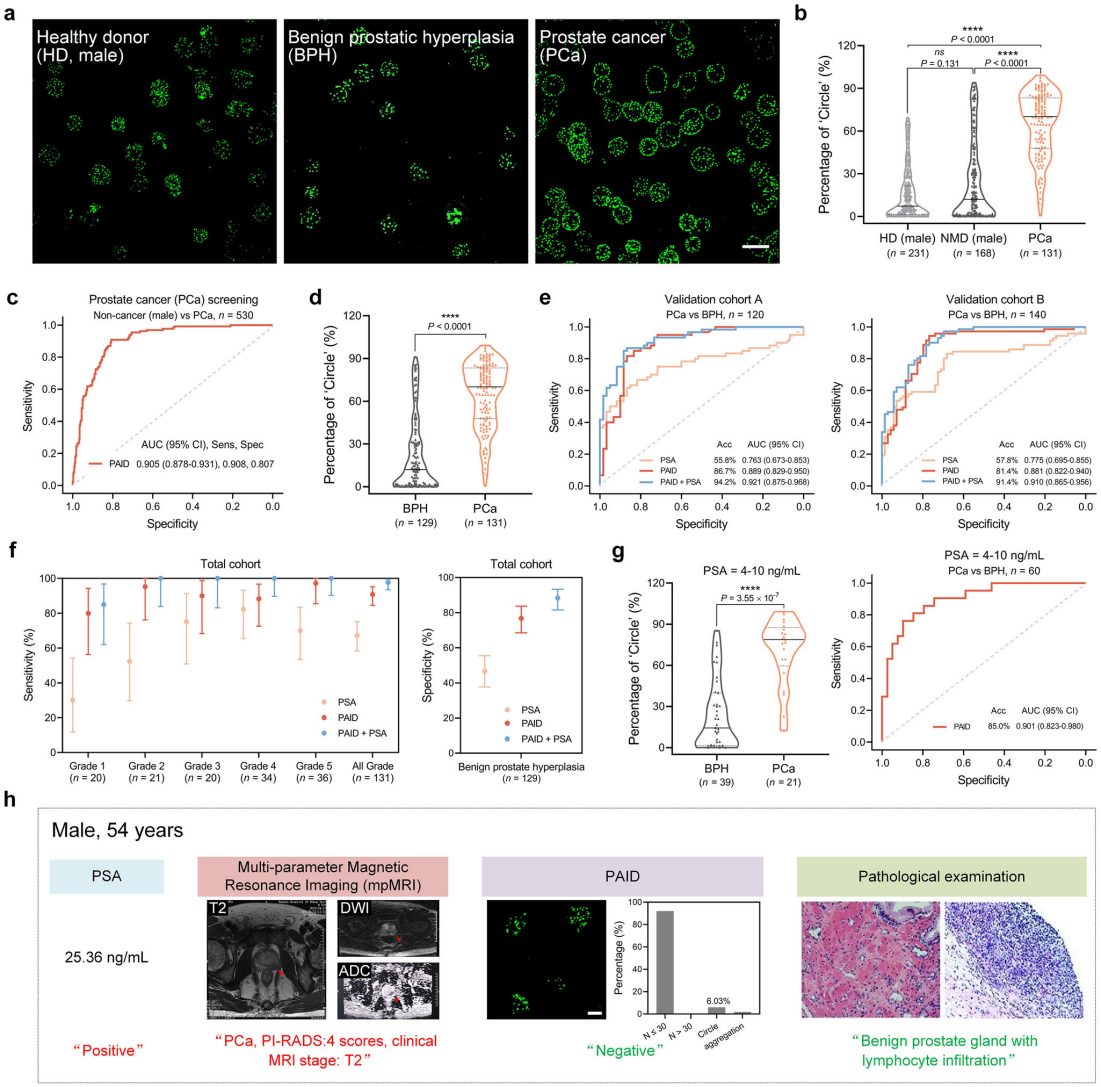
PAID outperforms clinical PSA in prostate cancer (PCa) screening and diagnosis. Violin plots show median (solid line) and interquartile range (IQR, dashed lines). For ROC analyses, AUC (95% CI) and accuracy (Acc) are indicated. a, Representative SIM images of α‐granule distributions in male HD (left, “N ≤ 30” pattern), benign prostatic hyperplasia (BPH, center, “N ≤ 30” pattern), and PCa (right, “Circle” pattern). Scale bars, 5 µm. b, “Circle” pattern percentages in HD (male), NMD (male), and PCa. The percentage of “Circle” pattern was significantly increased in PCa (Kruskal‐Wallis test with Bonferroni correction). c, ROC curve evaluates the performance of PAID in PCa screening. PAID showed robust performance in PCa screening. d, “Circle” percentage in BPH and PCa. The percentage of “Circle” pattern was significantly increased in PCa (Mann–Whitney *U* test). e, ROC curves evaluate the diagnostic performance of PAID, PSA, and their combinations for discriminating PCa from BPH in validation cohort A and cohort B. PAID and PAID combined with PSA showed improved performance (higher AUCs) compared to PSA alone. f, Sensitivity (left) and specificity (right) of PAID, PSA, and their combinations for detecting PCa across International Society of Urological Pathology (ISUP) grades. PSA was classified here according to clinical criteria: values > 10 ng/mL were considered indicative of cancer, whereas values < 4 ng/mL were considered non‐cancer. PAID generally improved sensitivity and specificity compared to PSA alone; combined use further enhanced diagnostic accuracy across ISUP grades. g, “Circle” percentage and ROC analysis for differentiating PCa from BPH in the PSA gray zone (4.0–10.0 ng/mL). h, Illustrative clinical case: A patient with benign prostate gland with lymphocyte infiltration diagnosed by pathology and correctly detected by PAID but initially misdiagnosed as PCa by PSA and MRI. Scale bars, 2 µm.

PCa is among the most prevalent malignancies afflicting the global male population. PSA (a commonly used clinical biomarker)‐based screening reduces PCa mortality but leads to a high false positive rate (60%–75%), resulting in a significant number of unnecessary biopsies and potential overdiagnosis and overtreatment [22, 23]. In order to address this critical need and ascertain the value of PAID in PCa screening, we included a total of 530 men (231 HD, 168 NMD, and 131 PCa patients) into the study. The results showed that the percentages of “Circle” pattern were significantly enhanced, averaging a 6‐fold increase in PCa patients (median: 70.1%), in comparison to both HD and NMD (median: 10.4%, *P* < 0.0001, Figure 2a,b), while there was no significant difference between HD and NMD (*P* > 0.05, Figure 2b). In the context of PCa screening, PAID exhibited a superior AUC value of 0.905 (95% CI 0.878–0.931) and a much lower false positive rate (19.3%) compared to PSA (60%–75%; Figure 2c). The optimal Youden index on the ROC curve indicated that PAID achieved a sensitivity of 90.8% and a specificity of 80.7% (Figure 2c).

Differentiating between PCa and benign prostatic hyperplasia (BPH) is clinically crucial due to their symptom overlap and divergent management strategies. Both conditions manifest as lower urinary tract symptoms, including urinary frequency, weak stream, and nocturia. Accurate differentiation is therefore essential for appropriate intervention and improved patient outcomes. PSA exhibits limited diagnostic value for PCa due to its moderate sensitivity (67.5%‐80%) and low specificity (∼40%) [24]. This constrained specificity arises because PSA is organ‐specific rather than cancer‐specific, and its levels are influenced by age, clinical factors, and benign conditions such as BPH [25]. To address this critical diagnostic challenge and further evaluate our PAID for differentiating PCa from BPH, we analyzed platelet α‐granule distributions from 131 PCa patients and 129 BPH patients. PCa patients displayed a significantly higher percentage of “Circle” pattern compared to age‐matched BPH controls (median: 70.1% vs 12.1%, *P* < 0.0001, Figure 2a,d, and Extended Data Figure S7a), despite a small overlap due to individual heterogeneity [26]. In the validation cohort A, PAID exhibited markedly higher accuracy (Acc, 86.7% vs 55.8%) and AUC (0.889, 95%CI 0.829–0.950 vs 0.763, 95% CI 0.673–0.853) compared to PSA (Figure 2e). The optimal Youden index on the ROC curve indicated that PAID achieved a sensitivity of 95.0% and specificity of 83.3% (Extended Data Figure S7b and Table S5). Furthermore, integrating PAID with PSA significantly boosted diagnostic accuracy to 94.2%, a marked 38.4% improvement over PSA alone (Figure 2e). Synergistically, combining PAID and PSA dramatically enhanced diagnostic performance: sensitivity rose from 66.7% to 98.3%, and specificity from 45.0% to 90.0% (Extended Data Figure S7b and Table S5). The excellent performance of PAID was maintained in the independent validation cohort B (Figure 2e, Extended Data Figure S7c and Table S5). Furthermore, in the total cohort, across all five grades of PCa, the sensitivity of PAID surpassed PSA (Figure 2f). And the combination of PAID with PSA yielded a substantial enhancement in overall sensitivity, rising from 67.2% to 97.7%, and a concomitant rise in specificity from 46.5% to 88.4% (41.9% improvement) (Figure 2f). Therefore, PAID markedly improves PCa diagnosis, surpassing PSA alone in accuracy, AUC, sensitivity, and specificity, and synergizes with PSA for enhanced performance. These findings establish PAID as a robust diagnostic approach for PCa, addressing PSA limitations in PCa diagnosis.

Distinguishing PCa from BPH in the PSA gray zone (typically 4.0–10.0 ng/mL) has long posed a challenge in urological clinical practice [27, 28, 29]. While PSA derivatives and composite markers like the Prostate Health Index and 4K score have aimed to improve PCa detection in this range, their inherent reliance on PSA fundamentally compromises reliability due to PSA's susceptibility to diverse clinical factors [30, 31]. To effectively address this diagnostic limitation, we evaluated the capacity of PAID to discriminate PCa from BPH within the PSA gray zone. We revealed that the percentage of “Circle” pattern was significantly elevated in PCa patients (median: 78.7%) compared to BPH patients (median: 14.3%) within the PSA gray zone (*P* < 0.0001, Figure 2g and Extended Data Figure S7d). PAID robustly discriminated PCa from BPH within the PSA gray zone, yielding high performance (accuracy: 85.0%, AUC: 0.901, 95% CI 0.823‐0.980) with 76.2% sensitivity, and 89.7% specificity (Figure 2g and Extended Data Table S5). Consequently, PAID resolves diagnostic uncertainty in the PSA gray zone, demonstrably surpassing PSA assessment.

Underdiagnosis and misdiagnosis remain significant challenges in the effective management of clinical PCa. PAID offers a promising avenue to mitigate these diagnostic shortcomings. Case 1 compellingly demonstrates PAID in enhancing diagnostic specificity, reducing false positives. A 54‐year‐old male patient presented with an elevated PSA level of 25.36 ng/mL and a Prostate Imaging Reporting and Data System score of 4, highly suggestive of PCa, T2 stage of clinical magnetic resonance imaging (MRI) (Figure 2h). However, PAID yielded a negative result (“Circle” pattern: 6.0%) (Figure 2h). Consistent with this, biopsy revealed BPH with lymphocytic infiltration (Figure 2h). This case highlights the capacity of PAID to refine diagnosis, preventing unnecessary interventions and overdiagnosis by accurately identifying benign conditions despite elevated PSA and suspicious imaging. Conversely, case 2 powerfully illustrates its potential in resolving diagnostic ambiguity in atypical PCa. A 64‐year‐old male presented with progressive dysuria and nocturia, and a persistently borderline PSA (initially 4.36 ng/mL, rising to 5.35 ng/mL) (Extended Data Figure S7e). Despite ineffective conventional medication and MRI suggesting BPH with chronic prostatitis, PAID revealed a strikingly elevated “Circle” pattern (83.3%), indicating PCa (Extended Data Figure S7e). Crucially, biopsy confirmed prostatic adenocarcinoma (Gleason 3 + 3 = 6, International Society of Urological Pathology (ISUP): grade 1), albeit in a small tissue volume (∼5%) (Extended Data Figure S7e). This case underscores that PAID can effectively detect PCa even when PSA and MRI findings are inconclusive, revealing early‐stagecancer missed by standard assessments.

### 2.3 PAID Augments Ovarian Cancer (OC) Screening, Diagnosis, and Recurrence Detection

**FIGURE 3:**
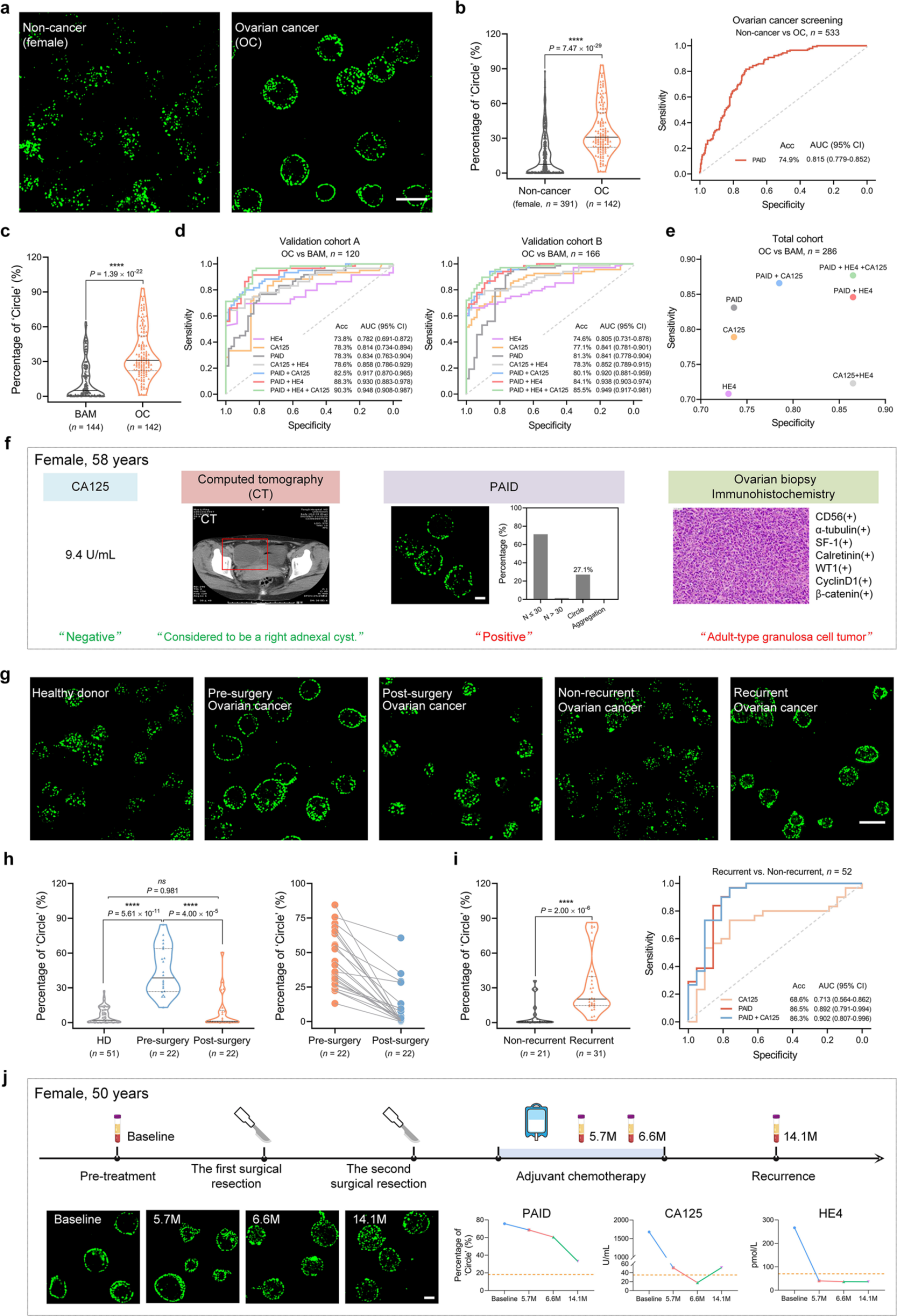
PAID outperforms current clinical biomarkers in ovarian cancer (OC) screening, diagnosis, and recurrence detection. Violin plots show median (solid line) and interquartile range (IQR, dashed lines). For ROC analyses, AUC (95% CI) and accuracy (Acc) are indicated. a, Representative SIM images of α‐granule distributions in female non‐cancer (left, “N ≤ 30” pattern) and OC (right, “Circle” pattern). Scale bar, 5 µm. b, “Circle” pattern percentage in female non‐cancer and OC patients. The percentage of “Circle” pattern was significantly increased in OC (Mann–Whitney *U* test). ROC curve evaluates the diagnostic performance of PAID in OC screening. PAID showed robust performance in OC screening. c, “Circle” pattern percentage in BAM and OC. d, ROC curves evaluate the diagnostic performance of PAID, CA125, HE4, and their combination for discriminating OC from BAM in validation cohort A and cohort B. PAID combined with HE4 and all biomarkers combined showed improved diagnostic performance (higher AUCs) compared to others. e, The sensitivity and specificity of PAID, CA125, HE4, and their combination for discriminating OC from BAM in total cohort. The combination of PAID with HE4, as well as the integration of all biomarkers, showed higher sensitivity and specificity compared to others. f, Illustrative clinical case: A patient with OC (adult‐type granulosa cell tumor) diagnosed by biopsy and correctly identified by PAID, but initially misdiagnosed as BAM based on CA125 and CT (red boxed region). Scale bar, 2 µm. g, Representative SIM images of α‐granule distributions in HD, OC patients with pre‐surgery, post‐surgery, non‐recurrent, and recurrent OC. Scale bar, 5 µm. h, “Circle” pattern percentage in HD, OC patients with pre‐surgery and post‐surgery. The percentage of “Circle” pattern was significantly decreased in post‐surgical OC (Wilcoxon signed‐rank test) and returned to HD level (Mann‐Whitney *U* test). i, “Circle” pattern percentage and ROC analysis for differentiating recurrent from non‐recurrent OC. PAID and PAID combined with CA125 showed enhanced diagnostic performance (higher AUCs) compared to CA125. j, Illustrative clinical case: Longitudinal monitoring of a patient who relapsed 14.1 months post‐treatment, using PAID, CA125, and HE4 at various time points: pre‐surgery, during adjuvant chemotherapy (5.7 and 6.6 months), and at recurrence (14.1 months). Scale bar, 2 µm.

The earlier detection of OC would significantly improve patient outcomes [32]. However, the benefit of screening using current methods, such as stand‐alone biomarker assays (e.g., CA125 and HE4) or longitudinal CA125 measurements in conjunction with transvaginal ultrasound (TVS), remains uncertain [32, 33, 34]. Consequently, no existing screening strategy has proven effective in identifying OC early enough to reduce mortality [34]. To address this critical diagnostic challenge, we evaluated the potential of PAID for OC screening. Remarkably, the percentages of “Circle” pattern were significantly enhanced, averaging a 4‐fold increase in OC patients (median: 31.0%, *n* = 142) compared to non‐cancer individuals (median: 7.4%; *P* < 0.0001, including HD and NMD, *n* = 391), with an AUC of 0.815 (95% CI 0.779‐0.852) (Figure 3a,b). Compared to CA125, PAID showed superior sensitivity (81.7% vs. 68.2‐77.1%) and positive predictive value (PPV, 51.8% vs. 21.7%) [35] (Extended Data Table S7). Although additional validation in fully independent datasets is warranted, the enhanced diagnostic capability represents a significant advancement in OC screening, potentially enabling earlier and more effective intervention. To this end, we are launching larger, prospective clinical trials to rigorously evaluate the screening efficacy of PAID.

OC diagnosis remains challenging due to the limited accuracy of current clinical methods, including TVS, CA125, and HE4, resulting in frequent false positives and unnecessary surgeries [36, 37]. Recent data indicate that only 15% of women undergoing surgery for suspected ovarian masses are ultimately diagnosed with OC, starkly highlighting the critical challenge in distinguishing OC from benign adnexal masses (BAM) [38]. In response to this critical need, we further investigated the diagnostic potential of PAID to differentiate OC from BAM. Analyzing α‐granule distributions in 144 BAM and 142 OC samples, we observed a significant divergence. Compared with BAM samples (median: 5.0%), OC samples showed a six‐fold enriched “Circle” pattern (median: 31.0%; *P* < 0.0001, Figure 3c). In the validation cohort A, PAID achieved accuracy comparable to CA125 and outperformed HE4 (Figure 3d). Strikingly, combining PAID with HE4 synergistically enhanced performance (accuracy: 88.3%, AUC: 0.930, 95% CI 0.883–0.978), surpassing other two‐biomarker combinations and matching the performance of the full biomarker panel (Figure 3d). Consistent with these findings, PAID maintained robust performance in the validation cohort B, mirroring CA125 and exceeding HE4 (Figure 3d). Notably, the combination of PAID and HE4 demonstrated superior performance (accuracy: 84.1%, AUC: 0.938, 95% CI 0.903–0.974) in validation cohort B, with no added benefit from CA125 inclusion (Figure 3d). In the total cohort, the combined PAID and HE4 demonstrated robust diagnostic performance, exhibiting a sensitivity of 84.6% and a specificity of 86.5% (Figure 3e and Extended Data Table S6). Critically, this combined approach significantly enhanced sensitivity by 5.7% and 13.8% over CA125 and HE4 alone, and specificity by 13% over either marker (Figure 3e and Extended Data Table S6). Furthermore, in the clinically challenging differentiation of OC from endometriosis [37], where CA125 is often confounded by non‐specific elevation, the PAID/HE4 assay demonstrated superior discriminatory power, exceeding CA125 and HE4 performance by 22.3% and 24.9%, respectively (Extended Data Figure S8). These collective findings establish PAID as a valuable adjunct to current biomarkers, significantly enhancing diagnostic accuracy for the critical differentiation of OC from BAM, with potential for improved clinical management.

PAID mitigates diagnostic uncertainty in complex gynecological pathologies, enhancing OC detection. In a clinically perplexing case of a 58‐year‐old woman presenting with reduced CA125 and computed tomography (CT) imaging suggestive of BAM, PAID accurately predicted an OC profile (Figure 3f). This prediction was subsequently verified by biopsy and immunohistochemistry, revealing an adult‐type granulosa cell tumor (Figure 3f). Similarly, in a 30‐year‐old woman with CA125 and TVS findings indicative of BAM, a borderline serous tumor, confirmed by biopsy, was concordantly identified by a malignant PAID profile (Extended Data Figure S9a). Moreover, PAID reliably distinguishes BAM. For instance, in a 29‐year‐old female with elevated CA125 and conflicting imaging results (TVS indicating benignity, CT raising malignancy concerns), PAID's accurate BAM profile aligned with the biopsy‐confirmed endometriosis diagnosis (Extended Data Figure S9b). Furthermore, a 39‐year‐old woman initially misdiagnosed with OC based on TVS and CA125 was correctly re‐classified as benign tubal mesosalpinx cyst by biopsy, a finding corroborated by PAID's BAM profile (Extended Data Figure S9c).

To further evaluate the therapeutic responsiveness of PAID in OC, we analyzed pre‐ and post‐surgery samples from 22 patients. Following tumor resection, a significant shift in α‐granule distribution patterns emerged. Specifically, “Circle” pattern significantly decreased in OC patients with post‐surgery. (*P* < 0.0001, Figure 3g and h). Consequently, “Circle” pattern in post‐surgical OC patients became statistically indistinguishable from that of healthy donors (*P* > 0.05, Figure 3g,h). These compelling findings definitively demonstrate the responsiveness of PAID to therapeutic intervention.

The suboptimal sensitivity of CA125 hinders the effective detection of recurrent ovarian cancer, thereby significantly compromising patient prognosis [40, 41]. To address this critical limitation, we evaluated the potential of PAID in detecting recurrent ovarian cancer. In a cohort of 31 recurrent and 21 non‐recurrent ovarian cancer patients, α‐granule distribution patterns revealed a recurrent ovarian cancer signature: a marked “Circle” pattern elevation (*P* < 0.0001, Figure 3g,i). PAID robustly identified recurrence with 86.5% accuracy and a high AUC of 0.892 (95% CI 0.791‐0.994), significantly surpassing CA125 (Figure 3i). Notably, CA125 addition did not improve accuracy (Figure 3i). PAID alone achieved 90.3% sensitivity and 81.0% specificity, representing a 30.3% sensitivity gain over CA125 (Extended Data Table S7). These data establish PAID as a robust and transformative tool for recurrent ovarian cancer, overcoming CA125 limitations.

Building on the established capacity of PAID to reflect treatment response and detect ovarian cancer recurrence, we evaluated its potential as an early predictor of recurrence. We enrolled 20 patients for prospective follow‐up; however, many were lost to follow‐up after receiving targeted or maintenance therapy at local hospitals and did not return for scheduled visits. Ultimately, only three patients completed long‐term monitoring, including one who experienced recurrence. In this recurrence case (Figure 3j), the “Circle” pattern remained elevated throughout treatment and was partially discordant with CA125 and HE4 dynamics, suggesting that PAID may provide complementary information not fully captured by standard CA125/HE4 monitoring; however, this observation remains exploratory and validation in larger cohorts is required. In addition, in two recurrence‐free individuals (follow‐up durations of 25.5 and 24.7 months, respectively), the percentages of “Circle” pattern normalized rapidly after treatment and remained stable throughout follow‐up, concordant with CA125 and HE4 dynamics (Extended Data Figure S10). Collectively, these longitudinal observations suggest the potential of PAID in dynamic monitoring, but its clinical utility for early recurrence warning requires confirmation in larger, longer‐term prospective cohorts with more complete follow‐up.

### 2.4 Tumor Cells Possibly Utilize Both Tumor‐Derived Exosomes and Proteins to Remodel Platelet α‐Granule Distributions

Despite prior evidence suggesting that tumor‐derived signals remodel platelet biology, research has primarily focused on alterations in platelet count, function, and RNA and protein profiles [13, 39, 42, 43, 44]. Consequently, the fundamental processes by which tumor cells alter platelet α‐granule distributions remain unclear.

To investigate the interactions between tumor cells and platelets, we utilized a widely used in vitro co‐incubation system [44, 45, 46]. This reductionist approach facilitates a focused analysis of the interactions between tumor cells and platelets, thereby offering insights into the cellular mechanisms responsible for tumor‐induced alterations in α‐granule distributions. Initially, we assessed the potential confounding effects of the tumor cell culture medium (RPMI 1640, at 37 °C) on α‐granule distributions. No significant changes were observed compared to the standard platelet storage condition over a 24 h period (ACDT, at 22°C; *P* > 0.05, Extended Data Figure S11a). Subsequently, we optimized the duration of co‐incubation and discovered that while the percentages of “Circle” pattern remained consistent for 6 h, there was a notable increase after 12 h (*P* < 0.001, Extended Data Figure S11b, and S11c), which justified selecting a 12 h co‐incubation period for our subsequent experiments.

**FIGURE 4:**
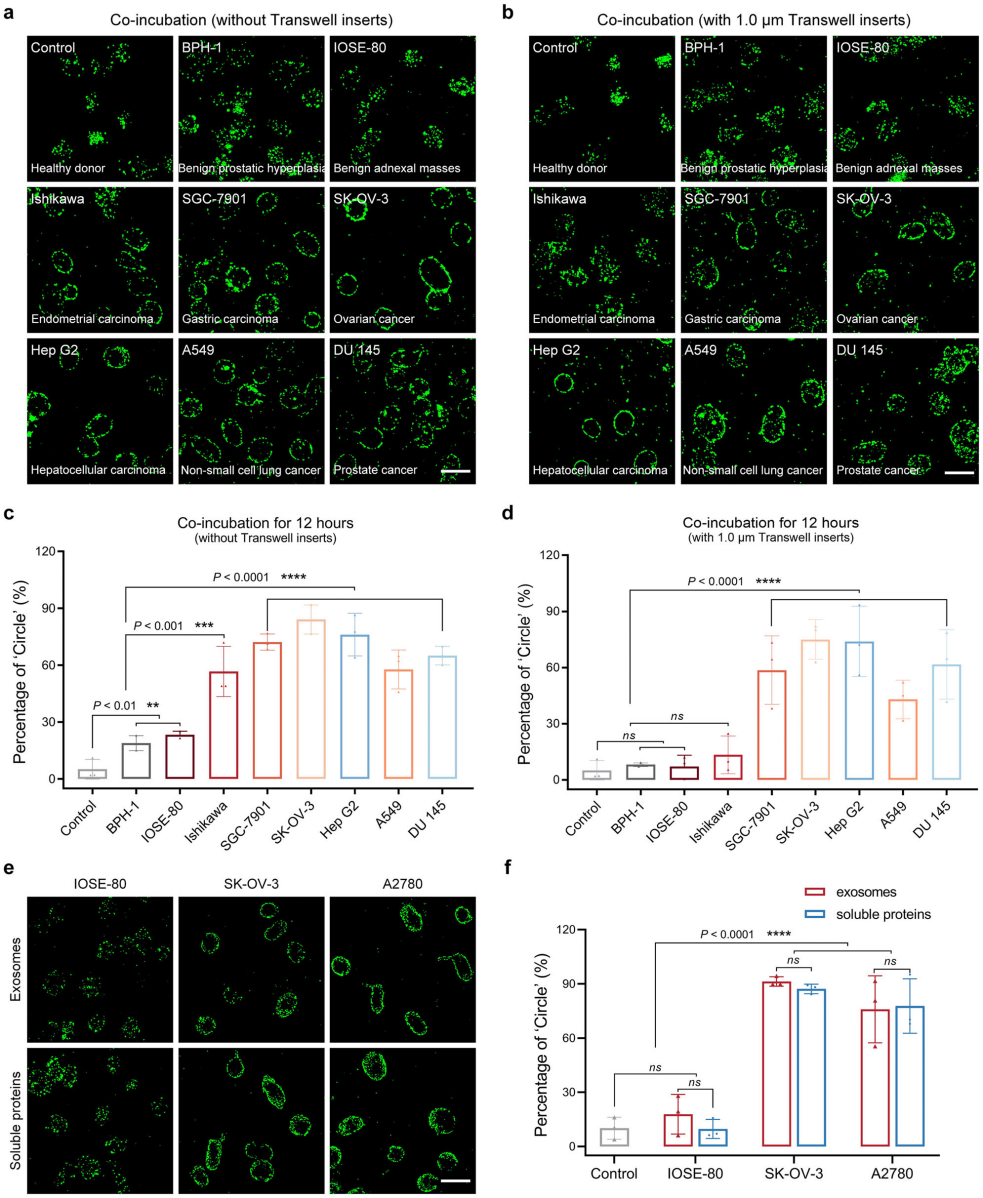
Platelet α‐granule distributions are reshaped by tumor‐derived exosomes and proteins. Control: platelets incubated without cells; Benign cell lines: BPH‐1 and IOSE‐80; Tumor cell lines: Ishikawa, SGC‐7901, SK‐OV‐3, Hep G2, A549, DU 145. Data are presented as mean ± s.d. (*n* = 3; the Student's *t*‐test for two groups or the one‐way ANOVA with Dunnett's post‐test for multiple comparisons for three or more groups). a‐d, Representative SIM images (a, b) and “Circle” pattern percentages (c, d) of α‐granule distributions in platelets after 12 h co‐incubation with tumor or benign cells. Co‐incubation with the clinical “Positive” tumor cell lines significantly increased “Circle” pattern percentages, regardless of Transwell separation, while direct co‐incubation with benign or clinical “Negative” tumor cell lines showed a moderate increase that was abolished by Transwell separation. e, f, Representative SIM images (e) and “Circle” percentage (f) of α‐granule distributions in platelets after being exposed to exosomes or soluble proteins from tumor (SK‐OV‐3, A2780) and benign cells (IOSE‐80). Both tumor‐derived exosomes and soluble proteins independently enhanced “Circle” pattern with comparable efficacy. Scale bars, 5 µm.

We then conducted co‐culture experiments involving platelets from HD and a diverse range of well‐characterized tumor cell lines, spanning different types of tumors and levels of malignancy, as well as benign control cells. Specifically, platelets were incubated for 12 h with two benign cell lines, BPH‐1 (benign prostate hyperplasia) and IOSE‐80 (benign adnexal masses), one clinical “Negative” tumor cell line (defined as tumor cell lines derived from cancer types that showed no significant increase in the “Circle” pattern in patient cohorts), Ishikawa (endometrial carcinoma), and five clinical “Positive” tumor cell lines (defined as tumor cell lines derived from cancer types where patient samples exhibited significantly higher “Circle” proportions than non‐cancer controls), including SGC‐7901 (gastric cancer), SK‐OV‐3 (ovarian cancer), Hep G2 (hepatocellular carcinoma), A549 (non‐small cell lung cancer), and DU 145 (prostate cancer) (Figure 4a). Tumor cells typically interact with platelets through both direct cell‐cell contact and indirect interactions involving secreted factors. Therefore, in addition to direct co‐incubation, we also used Transwell inserts to simulate indirect contact by separating platelets from the cell lines while allowing the exchange of secreted factors. Notably, consistent with clinical observations (Figure 1e), co‐incubation of platelets with these “Positive” tumor cell lines markedly enhanced the percentages of “Circle” pattern, whether or not physically separated by a Transwell membrane (*P* < 0.0001, Figure 4a–d). Intriguingly, while direct co‐incubation of both benign and the “Negative” tumor cells induced a moderate increase in “Circle” pattern, this effect was abolished by physical separation via Transwell inserts (Figure 4a–d). Additionally, in situ analysis revealed “Circle” patterns in platelets adjacent to both benign and malignant tumor cells (Extended Data Figure S12). These results imply that direct co‐incubation of platelets with tumor cells, whether benign or malignant, can induce “Circle” pattern. However, the enhanced “Circle” pattern observed through indirect interactions appears to be specific to these “Positive” tumor cell lines, consistent with the clinical observations (Figure 1e). Given the physiological reality in patients, platelets are more likely to be exposed to tumor‐derived factors within the bloodstream rather than directly contacting tumor cells. Accordingly, we suggest that alterations in platelet α‐granule distributions primarily stem from indirect interactions with tumor‐derived factors in the bloodstream. Additionally, we assessed the reversibility of the “Circle” phenotype by transferring platelets after their initial co‐incubation with malignant tumor cells to a subsequent co‐incubation with benign cell lines. This treatment did not reduce the proportion of “Circle” platelets (Extended Data Figure S13), indicating that the phenotype is not readily reversible.

Tumor‐derived components, including exosomes and soluble proteins, are key mediators of indirect cell communication [12, 13, 47, 48]. To determine which of these components modulate platelet α‐granule distributions, we incubated platelets with either concentrated exosomes (Extended Data Figure S14) or soluble proteins isolated from tumor cell‐conditioned media. Notably, both tumor‐derived exosomes and soluble proteins independently enhanced “Circle” pattern with comparable efficacy (Figure 4e,f). These findings suggest that tumor cells utilize both exosome and soluble protein pathways to remodel platelet α‐granule distributions, and that the “Circle” phenomenon is likely not triggered by a single factor but rather arises from the synergistic action of multiple factors, thereby providing a new perspective on how tumor cells influence platelets. Unfortunately, mouse models are severely limited for exploring the underlying biological processes in our study, because our method requires 2 mL of peripheral blood per experiment, whereas the total circulating volume of an adult mouse is only 1.5–2.0 mL and institutional guidelines restrict any single withdrawal to approximately 10 % of that volume (∼ 0.18 mL) within 24 h. Given the complex composition of these exosomes and soluble proteins and the limited prior mechanistic investigation into this phenomenon, fully elucidating the precise molecular pathways is a complex undertaking that demands considerable time and effort. Our efforts to identify these factors and elucidate their synergistic mechanisms are thus underway.

## 3 Discussion

Previous studies on platelets, exploring them as a compelling alternative for circulating tumor components, have primarily focused on their RNA and protein signatures [42, 49, 50]. Although promising, these approaches have also encountered several hurdles, such as limited sensitivity to tumor‐derived signals and challenges in data interpretation [10, 51]_._ Organelle‐level alterations in platelets remain largely unexplored due to their extremely small size, being merely ∼1/300 the volume of a HeLa cell [16, 19, 52, 53]. A cryo‐electron tomography study of a small ovarian cancer cohort (12 patients and 11 controls) revealed morphological differences in mitochondria and microtubules between patients and controls, suggesting the potential of these features as diagnostic biomarkers [16]. However, the clinical translation of these findings has been hindered by the inherent low throughput of electron microscopy [19]. To overcome this limitation, we previously developed a SIM‐based imaging workflow that enables high‐throughput, super‐resolution visualization of platelet subcellular structures with exceptional clarity. This workflow also includes a rigorously standardized protocol for platelet sample preparation that encompasses all steps from blood collection to platelet isolation, and immunofluorescence staining, thus minimizing artifacts introduced by in vitro factors such as anticoagulants, storage conditions, and fixation reagents. Using this workflow, we systematically analyzed the key subcellular structures in platelets, including mitochondria, dense granules, α‐granules, and microtubules and notably, we identified, for the first time, cancer‐associated alterations in α‐granule distribution [15]. Nevertheless, due to the limited sample size (n = 144 patients across five cancer types, with only a few to ∼10 cases per type), critical questions remain: the robustness and validity of these alterations in larger patient cohorts, their clinical relevance across diverse cancer types, and the underlying mechanisms have yet to be established.

To bridge these gaps and comprehensively evaluate clinical utility, we first substantially extended our cohort by undertaking an unprecedented multicenter study of 1,556 individuals spanning nine cancer types and twelve non‐malignant diseases. Our results demonstrate that cancer‐associated alterations in α‐granule distributions (particularly the increased “Circle” pattern) are a reproducible population‐level cancer phenotype, establishing our PAID as a promising non‐invasive tool for cancer screening and diagnosis with marked efficacy in prostate, ovarian, hepatocellular carcinoma, and non‐small cell lung cancers. These findings advance platelet α‐granule morphology from an exploratory concept to a viable potential diagnostic modality.

To further validate its clinical utility, we investigated the performance of PAID in prostate cancer (PCa) to address the primary limitations of PSA testing across the diagnostic pathway. For screening, PAID improves the poor specificity of PSA, demonstrating a superior AUC value of 0.905 (95% CI 0.878‐0.931) and reducing the false‐positive rate to 19.3% from the typical 60.0%–75% for PSA [20]. This enhanced specificity is critical for mitigating the risk of over‐diagnosis. For diagnosis, integrating PAID with PSA enhanced the overall accuracy of differentiating PCa from benign prostatic hyperplasia (BPH) to 94.2%, a 38.4% increase over PSA alone. Crucially, within the challenging PSA gray zone, PAID achieved an exceptional 85.0% accuracy, directly addressing a critical unmet need in urological practice. Current diagnostic strategies are notoriously limited within this PSA gray zone, often leading to diagnostic uncertainty and unnecessary biopsies [22, 28]. Collectively, these findings establish PAID as a non‐invasive tool to refine the PCa diagnostic pathway, offering a clear, quantitative basis to guide biopsy decisions, minimize patient harm, and reduce the substantial burdens associated with diagnostic uncertainty.

We further showcased the versatile applicability of PAID in the screening and diagnosis of ovarian cancer (OC). In OC screening, PAID sets a new performance benchmark with significantly enhanced sensitivity (81.7% vs 68.2%–77.1%) and positive predictive value (PPV, 51.8% vs 21.7%) over CA125 [35]. This dual improvement is critical for patients: higher sensitivity reduces the risk of missing early‐stage disease, while higher PPV minimizes the anxiety and unnecessary procedures stemming from false‐positive results. Furthermore, the combination of PAID with HE4 for differentiating OC from benign adnexal masses achieved impressive sensitivity (84.6%) and specificity (86.5%). Compared to current clinical practice relying on solitary markers like CA125 or HE4, our combined approach markedly enhances sensitivity by 5.7% and 13.8% alone, and specificity by 13% over either marker [35]. Strikingly, in the challenging differential diagnosis between OC and endometriosis [37, 54], combining PAID with HE4 analysis achieved diagnostic accuracy substantially surpassing that of CA125 and HE4 alone, yielding remarkable gains exceeding 22.3% and 24.9%, respectively. These findings reveal the powerful synergy between PAID and established OC biomarkers, offering a transformative strategy to enhance diagnostic precision, particularly in challenging differential diagnoses [38, 55], and ultimately revolutionize clinical decision‐making in OC management.

Moreover, in the cross‐sectional analysis of patients with recurrent ovarian cancer, PAID demonstrated a sensitivity of 90.3% and a specificity of 81.0%, representing a 30.3% absolute gain in sensitivity over CA125. These findings suggest that PAID may serve as a complementary biomarker for detecting recurrence. During the longer‐term prospective monitoring, unfortunately, only three of the 20 enrolled patients completed the follow‐up. Nevertheless, we observed an increased proportion of “Circle” pattern, which may indicate early recurrence; however, this observation remains exploratory and requires validation in larger cohorts.

Finally, our results show that tumor cells possibly utilize both tumor‐derived exosomes and proteins to remodel the spatial organization of platelet α‐granules, thereby driving an increased prevalence of the diagnostically relevant “Circle” pattern. While tumor‐derived signals are known to remodel platelet biology (e.g., RNA and proteins) [13, 42, 43, 44], our findings demonstrate a novel possible dimension of this systemic response. However, due to the complex composition of these exosomes and soluble proteins, the precise molecular mechanism remains to be fully elucidated.

Several aspects warrant further investigation. First, while our study included a diverse cohort of non‐malignant disease types, future studies will benefit from expanding the cohort to encompass individuals with chronic inflammation, cardiovascular diseases, cancer‐prone populations, and a broader spectrum of cancer types, enabling an even more comprehensive evaluation of PAID diagnostic performance. Although stringent inclusion criteria were adopted to minimize major confounders, the retrospective nature of clinical data collection limited comprehensive assessment of comorbidity profiles; consequently, unmeasured covariates remain a potential source of bias. Future studies should therefore prospectively collect detailed comorbidity data, especially for patients with comorbid cancers and chronic diseases, to enable multivariable adjustment and validate the independent predictive value of PAID. Second, although SIM provided the necessary resolution for this study, reliance on this expensive, low‐throughput technique remains a significant barrier to clinical translation. Nevertheless, we anticipate that as the unique value of this method is increasingly validated across clinical scenarios, it will spur the development of more efficient, lower‐cost, and higher‐throughput imaging systems tailored to this specific assay. We are actively collaborating with instrument developers to optimize the imaging system and reduce costs, although this represents a long‐term effort. Meanwhile, rapid advances in computational super‐resolution algorithms, ongoing hardware innovations, and broader community engagement will collectively help overcome this bottleneck. Third, further elucidation of the precise molecular pathways of tumor cells governing the α‐granule distribution alternations is needed to fully appreciate its functional implications in cancer biology and progression.

In summary, by expanding our cohort to over 1,500 participants across nine major cancer types and twelve non‐malignant conditions, encompassing a broad spectrum of benign tumors and inflammatory states, we demonstrated robust cancer‐associated alterations in α‐granule distribution patterns, establishing PAID as a new potential paradigm for non‐invasive liquid biopsy. Through comparison and integration with established clinical diagnostic markers, we identified clinical applications suited to our method, representing substantial progress toward clinical translation. Our strategy leverages the inherent abundance and accessibility of platelets rather than relying on rare circulating tumor‐derived components, offering a promising new avenue for cancer management.

## 4 Methods

### 4.1 Ethics Approval and Study Population

This study was conducted in accordance with the principles of the Declaration of Helsinki. Ethical approval was obtained from the ethics committee of each participating hospital, namely: Tongji Medical College, Huazhong University of Science and Technology (Approval Numbers: 2021‐S225, 2024‐S116 and 2024‐S245), Renmin Hospital of Wuhan University (Approval Number: WDRY2024‐K054). The requirement for informed consent was waived by the ethics committee because this observational study used residual blood samples collected after routine clinical testing and involved minimal risk to participants.

A total of 1,556 participants were enrolled in this study from the Wuhan Blood Center and four hospitals. These participants included 342 healthy donors (HD), 448 non‐malignant diseases patients (NMD, > 12 disease types), and 766 patients with cancer (9 cancer types). The participating hospitals were: Tongji Hospital Affiliated to Tongji Medical College, Huazhong University of Science and Technology; Union Hospital Affiliated to Tongji Medical College, Huazhong University of Science and Technology; Renmin Hospital of Wuhan University; and Traditional Chinese and Western Medicine Hospital of Wuhan Affiliated to Tongji Medical College, Huazhong University of Science and Technology. Inclusion criteria of this research include: (1) healthy donors confirmed by clinical examination and laboratory tests to have no malignancies, severe systemic diseases, or hematological abnormalities; patients with histologically or clinically confirmed benign, non‐malignant diseases; patients with cancer pathologically confirmed by surgical or biopsy specimens; (2) participants who had not received surgery, chemotherapy, radiotherapy, or immunotherapy before blood sampling; and (3) participants who had not taken aspirin, clopidogrel, or other antiplatelet/anticoagulant drugs within two weeks prior to blood collection. Exclusion criteria are: (1) individuals with a history or presence of diabetes mellitus and hematological disorders; (2) individuals who had received corticosteroids, immunosuppressive agents, or cytotoxic drugs within two weeks before blood collection; and (3) individuals who experienced acute thrombotic or major hemorrhagic events within three months prior to enrollment. For the follow‐up group, 22 patients with ovarian cancer were re‐enrolled at least one month after surgery. In addition, 3 ovarian cancer patients were re‐enrolled three times: twice during chemotherapy and once at least six months after the last treatment. Furthermore, 31 patients with recurrent ovarian cancer and 21 patients without recurrent ovarian cancer were enrolled to evaluate the performance of indicators in detecting recurrent ovarian cancer.

The cancers included in the study were: myeloproliferative neoplasm (MPN), cervical carcinoma (CC), endometrial carcinoma (EC), gastric carcinoma (GC), ovarian cancer (OC), non‐small cell lung cancer (NSCLC), hepatocellular carcinoma (HCC), cholangiocarcinoma (CCA), and prostate cancer (PCa). The non‐malignant diseases included in the study were: ovarian cyst, fallopian tube cyst, chronic salpingitis, uterine myoma, endometrial polyps, endometrial hyperplasia, uterine prolapse, cervical intraepithelial neoplasia grade III, benign pulmonary nodule, benign prostatic hyperplasia, cirrhosis of the liver and hepatic hemangioma. The benign adnexal masses encompassed ovarian cyst, fallopian tube cyst and chronic salpingitis.

Clinical information, such as age, gender, and pathological diagnosis, and tumor stage, was obtained from medical records and is presented in Extended Data Table S1.

### 4.2 Sample Collection, Processing and Immunofluorescence Staining

Blood samples were collected in 2 mL or 4 mL purple‐capped Vacutainer tubes containing the anticoagulant ethylenediaminetetraacetic acid (EDTA). Samples were stored at room temperature and processed within 24 h of collection using the standardized platelet sample processing procedure, as described previously [15].

First, platelet‐rich plasma (PRP) was isolated from whole blood by centrifugation at 200 × *g* for 12 min. The PRP was then diluted 1:5 in dilution buffer. The buffer consisted of 10% acid citrate dextrose (ACD; Nigale Biotechnology, China, cat. no. H20053787) and 90% Tyrode's‐4‐(2‐hydroxyethyl)‐1‐piperazineethanesulfonic acid (Tyrode's‐HEPES) buffer. Tyrode's‐HEPES buffer contained 68.92 mmol sodium chloride (NaCl), 1.45 mmol potassium chloride (KCl), 0.50 mmol magnesium chloride (MgCl_2_), 0.27 mmol sodium dihydrogen phosphate (NaH_2_PO_4_), 6.01 mmol sodium bicarbonate (NaHCO_3_), 2.75 mmol glucose, and 5.00 mmol HEPES, pH 7.2. To minimize the impact of centrifugation and subsequent processing on platelet subcellular structures, samples were incubated for 2 h at 37°C. Next, platelets were fixed at room temperature for 30 min by adding an equal volume of 8% paraformaldehyde (PFA, Sigma‐Aldrich, cat. no. P6148) in PHEM buffer. PHEM buffer contained 30.00 mmol piperazine‐N, N'‐bis (2‐ethanesulfonic acid) (PIPES), 12.50 mmol HEPES, 5.00 mmol ethylene glycol‐bis (β‐aminoethyl ether)‐N, N, N′, N′‐tetra acetic acid (EGTA), 1.00 mmol MgCl_2_ in a 500 mL system, pH 7.0). Finally, the fixed platelets were washed three times with Phosphate Buffered Saline (PBS) by centrifugation at 1500 × *g* for 3 min per wash, and then stored at 4°C for further use.

For immunofluorescence staining of platelet α‐granules, the platelets were first seeded onto dishes treated with 0.01% poly‐L‐lysine, followed by three washes with PBS. The platelets then underwent a series of processing steps: permeabilization with 0.2% polyethylene glycol tert‐octylphenyl ether (Triton X‐100) for 10 min; blocking with blocking buffer (10% normal goat serum (Beyotime, Beyotime Biotechnology, China, cat. no. C0265), 1% Bovine Serum Albumin (BSA, Biofroxx, Neofroxx, Germany, cat. no. 143 183), and 0.05% Triton X‐100) for 1 h; staining with rabbit polyclonal anti‐human von Willebrand factor antibodies (Dako, Dako North America Incorporated, US, cat. no. A0082, 1:1000 dilution, RRID: AB_2315602) for 4 h at room temperature or 12 h at 4°C; staining with Alexa Fluor 488‐conjugated goat anti‐rabbit IgG (Abcam, Abcam, UK, cat. no. ab150077, 1:500 dilution, RRID: AB_2630356) for 1 h at room temperature; and post‐fixation with 4% PFA in PBS for 10 min. The prepared platelet α‐granule samples were stored at 4°C until imaging.

### 4.3 Image Collection and Processing

Images of platelets were captured using two structured illumination microscopes: a Multi‐SIM (NanoInsights‐Tech, Guangzhou, China) equipped with a 100×/1.49 NA oil immersion objective (Nikon) and a HiS‐SIM (Guangzhou Chaoshiji Biotechnology, Guangzhou, China) equipped with a 100×/1.50 NA oil immersion objective (Olympus). Both bright‐field and fluorescence images of platelets were acquired. SIM fluorescence images were obtained using 488 nm laser light and reconstructed using SI‐Recon software (Multi‐SIM) or Reconstruction software (HiS‐SIM).

Standard operating procedure (SOP) for image acquisition: Image acquisition was performed using the multi‐region‐of‐interest (Multi‐ROI) mode of the SIM system, employing a 3 × 3 tiling array (nine adjacent fields). The focal plane was determined on the central tile and then locked for the entire array, after which the system automatically acquired a total of 9 images (each 2048 × 2048 pixels; pixel size = 30.6 nm) covering the surrounding area. To guarantee representative sampling, a cumulative total of 500–800 single‐dispersed platelets was recorded per participant, exceeding the statistically determined minimum of 200 platelets that has been shown to stabilize morphological pattern statistics^15^. Platelet density was assessed by randomly selecting 2–3 fields of view across the dish and counting single, dispersed platelets in each field. The number of imaging positions was then adjusted according to the observed density: nine, six, or four non‐overlapping positions per dish—corresponding to 6–9, 10–15, or 16–20 platelets per field—were randomly chosen, and a full 3 × 3 image array (9 images) was acquired at each position. In most experiments the density fell within 10–15 platelets per field, so six positions (54 images) were recorded most frequently. In total, approximately 540–810 platelets were imaged per dish, satisfying the predefined statistical criterion. To assess data reproducibility and inter‐operator variability, six samples were repeatedly imaged by six independent operators from different centers, all adhering to the SOP. The inter‐operator coefficients of variation ranged from 1.30 % to 7.74 % (Extended Data Figure S15). A variance‐component analysis showed that 54.79 % of the total variance was attributable to between‐sample variability, 5.68 % to operator‐related variability, and 39.53 % to residual error. Thus, data acquired with the SOP are reproducible across operators, with between‐sample variability—rather than inter‐operator variability—being the main source of variation.

The acquired images (approximately 50 images, containing more than 500 platelets) were then analyzed using a previously developed automated image analysis workflow [15]. This workflow included a convolutional neural network (CNN) model based on ResUNet for cell segmentation and a CNN model based on the ResNet‐50 framework for classifying platelet α‐granule distribution pattern. Complete architectural details, training protocols, and original validation performance of the models are provided in Supplementary Material, Section S1. All model architectures and parameters were fixed, and no fine‐tuning or retraining was performed. Using these fixed models as analytical tools, the diagnostic performance of PAID was independently evaluated in two separate clinical cohorts.

Validation cohort A comprised 120 patient cases and was used for the first independent performance evaluation. The required sample size was estimated a priori using PASS software (version 15.0). Based on a two‐sample t‐test allowing unequal variances, with a two‐sided significance level of 0.05 and 90% statistical power, we calculated the minimum required sample size. Considering both the minimum sample size requirements and the overall availability of cases, we set the size of validation cohort A to 120 participants, including 60 benign and 60 malignant cases. Validation cohort B comprised an additional ≥ 120 non‐overlapping patient cases and was used for a second independent performance evaluation to assess the robustness and generalizability of the model. To ensure the robustness of the findings and minimize selection bias, all eligible patients, including both benign and malignant cases, were randomly assigned to one of two validation cohorts using a predefined allocation scheme. A random sequence generated by SPSS version 26.0 was used to allocate participants. Validation cohort A was designed to include exactly 60 malignant patients and 60 benign controls, with the specific individuals selected entirely at random. All remaining patients were then assigned to validation cohort B.

Following image analysis, the number of α‐granules with the indicated distributions (N ≤ 30, N > 30, Circle, and Aggregation), as well as their percentages, were obtained. These output data were then subjected to statistical analysis using SPSS to plot ROC curves, calculate the Area Under the Curve (AUC) with confidence intervals, and determine the cutoff value for each cohort. The stability of the model's performance was assessed by directly comparing the AUC values and optimal thresholds between the two independent cohorts.

### 4.4 Cell Culture

Ten different human cell lines were used in this study. These cell lines included: Human Endometrial Cancer Cell Line Ishikawa (cat.# CL‐0283, RRID: CVCL_2529), Human Gastric Adenocarcinoma Cell line SGC‐7901 (cat.# CL‐0206, RRID: CVCL_0520), Human Hepatocellular Carcinoma Cell line Hep G2 (cat.# CL‐0103, RRID: CVCL_0027), Human Non‐Small Cell Lung Cancer Cell Line A549 (cat.# CL‐0016, RRID: CVCL_0023), Human Ovarian Carcinoma Cell line A2780 (cat.# CL‐0013, RRID: CVCL_0134), Human Ovarian Carcinoma Cell line SK‐OV‐3 (cat.# CL‐0215, RRID: CVCL_0532), Human Prostate Cancer Cell Line PC‐3 (cat.# CL‐0185, RRID: CVCL_0035), Human Prostate Cancer Cell Line DU 145 (cat.# CL‐0075, RRID: CVCL_0105), Human Benign Prostatic Hyperplasia Cell Line BPH‐1 (cat.# CL‐0865, RRID: CVCL_1091), and Human Normal Ovarian Epithelial Cell Line IOSE‐80 (cat.# CTCC‐400‐0117, Zhejiang Meisen Cell Technology Co., Ltd, RRID: CVCL_5546). Cell lines were purchased from Wuhan Pricella Biotechnology Co., Ltd unless otherwise noted.

A549, SGC‐7901, Hep G2, PC‐3, BPH‐1 and IOSE‐80 were cultured in Roswell Park Memorial Institute 1640 medium (RPMI 1640, Pricella) supplemented with 10% fetal bovine serum (FBS, Gibco) and 1% antibiotic‐antimycotic. The remaining cell lines (Ishikawa, A2780, SK‐OV‐3 and DU 145) were cultured in Dulbecco's Modified Eagle Medium (DMEM, Pricella) supplemented with 10% FBS and 1% antibiotic‐antimycotic. All cells were maintained at 37°C with 5% CO_2_ in a humidified atmosphere (Thermo Fisher Scientific).

### 4.5 Isolation of Exosomes and Free Proteins

Cells were seeded in 100‐mm diameter cell culture dishes and cultured in growth medium (DMEM and RPMI 1640 supplemented with 10% FBS) to 80% confluency. To induce exosome secretion, the cells were then washed three times with PBS and cultured for 24 h in fresh RPMI 1640 medium (without FBS). Conditioned media from ten dishes (approximately 70 mL) was collected and subjected to sequential centrifugation at 4°C to remove cell debris and larger vesicles: first at 2,000  × *g* for 10 min, then at 10,000 × *g* for another 10 min. The supernatant was then ultracentrifuged at 100,000 × *g* for 70 min at 4°C. The resulting exosome pellet was washed again with PBS via ultracentrifugation and resuspended in 350 µL of PBS. The free proteins in the remaining exosome‐depleted supernatant were then concentrated and purified to 0.5 mL using a BS‐UFC‐150‐010 10 kDa ultrafiltration unit by centrifugation at 4°C and 5000 × *g* for 10 min.

### 4.6 Characterization of Exosomes

The morphology of the exosomes was examined using transmission electron microscopy (TEM). The size and concentration of the exosomes were measured by nanoparticle tracking analysis (NTA). The expression of Cluster of Differentiation 9 (CD9), Histone H3, and Tumor Associated Glycoprotein 101 (TSG101) on the surface of exosomes was verified using Western blotting.

### 4.7 Western Blot

The isolated exosomes were separated by 10% sodium dodecyl‐sulfate polyacrylamide gel electrophoresis (SDS‐PAGE) and transferred to a polyvinylidene difluoride (PVDF) membrane (Millipore, catalog# IPVH00010). The membranes were then processed as follows: First, membranes were blocked with 5% BSA in Tris‐buffered saline‐Tween 20 (TBST; 0.2 M Tris [pH 7.4], 1.5 M NaCl, 0.1% thimerosal, and 0.5% Tween 20) for 2 h. Next, membranes were incubated overnight at 4°C with primary antibodies against CD9 (catalog# ab263019, Abcam, RRID: AB_3076464), Histone H3 (catalog# ab176880, Abcam, RRID: AB_2751009) and TSG101 (catalog# ab125011, Abcam, RRID: AB_10974262) at a 1:5,000 dilution. Subsequently, membranes were incubated for 1 h at room temperature with horseradish peroxidase (HRP)‐conjugated secondary antibodies (Amersham, catalog# A0208 and A0216) at a 1:5,000 dilution. Finally, membranes were developed with an enhanced chemiluminescence (ECL) kit (Amersham, catalog# 32132) and band intensities were quantified using ImageJ (RRID: SCR_003070).

### 4.8 Coculture of Platelet with Cells, Exosomes and Free Proteins of Cancer and Benign Cell Lines

Cells were seeded in confocal dishes or 24‐well plates using growth medium (DMEM or RPMI 1640 supplemented with 10% FBS) and cultured to 80% confluency. The cells were then washed three times with PBS, followed by the addition of a specified volume of platelet‐rich plasma (PRP; 8 × 10^11 platelets/mL) and fresh RPMI 1640 medium (without FBS): Co‐incubation (without Transwell inserts), 100 µL of PRP and 600 µL of RPMI 1640 medium were added to 24‐well plates; Co‐incubation (without Transwell inserts, direct interaction), 100 µL PRP and 500 µL RPMI 1640 medium were added to confocal dishes; Co‐incubation (with 1.0 µm Transwell inserts), 100 µL PRP and 600 µL RPMI 1640 medium were added to 24‐well plates equipped with 1.0 µm Transwell inserts; this configuration physically separated platelets and cells while permitting soluble factor exchange.

All cell‐platelet interaction experiments were incubated at 37°C for the indicated times (0, 6, 12, or 18 h). Following incubation, cells and platelets were fixed with 4% PFA for 30 min. The fixed cells were then washed three times with PBS (1500 × *g*, 3 min per wash) and resuspended in 1 mL PBS. Immunofluorescence staining of platelet α‐granules was performed as described previously. All cell‐platelet interaction experiments were performed in triplicate.

Separately, the co‐culture of exosomes/ free proteins and platelets was performed by adding 500 µL fresh RPMI 1640 medium (without FBS), 100 µL PRP (8 × 10^11 platelets/mL), and 100 µL of isolated exosomes/free proteins to 24‐well plates (without cells) sequentially. The co‐culture was incubated at 37°C for 12 h. The platelets were then fixed and stained as described above. All coculture experiments were performed in triplicate.

### 4.9 Statistical Analysis

Clinical information and percentages of platelets with the indicated α‐granule patterns are presented as mean ± standard deviation (SD) or median [interquartile range (IQR)].

The differences in the percentages of platelets with indicated α‐granule patterns were assessed using the following statistical tests: for paired samples (e.g., before and after surgery), the Wilcoxon signed‐rank test; for unpaired samples, the Mann‐Whitney *U* test (for two groups) or the Kruskal‐Wallis test with Bonferroni correction for multiple comparisons (for three or more groups). The differences in “Circle” percentage of α‐granule in platelets after co‐incubation with tumor or benign cells were assessed using the following statistical tests: the Student's *t*‐test (for two groups) or the one‐way ANOVA with Dunnett's post‐test for multiple comparisons (for three or more groups). All significance tests were two‐tailed. Statistical significance was defined as *P < 0.05, **P < 0.01, ***P < 0.001, and ****P < 0.0001. Non‐significant differences are indicated by “ns”.

The diagnostic performance of platelet α‐granules was evaluated using ROC curve analysis, including the area under the curve (AUC), 95% CI, Youden index, sensitivity, specificity, positive predictive value (PPV), negative predictive value (NPV), and accuracy (Acc). The cutoff value was determined by the maximum Youden index. To evaluate the diagnostic performance of the combined biomarkers (e.g., PAID + CA125, PAID + HE4, etc.), a multivariable binary logistic regression model was fitted using SPSS (RRID: SCR_002865) version 26.0. The dependent variable was the patient's histological diagnosis, coded as 1 for cancer and 0 for non‐cancer. The independent variables were the raw concentration values of the biomarkers included in each specific combination. CA125, HE4, and PSA were included as continuous variables. The PAID biomarker, expressed as a percentage (ranging from 0 to 100), was also treated as a continuous predictor. All variables were entered into the model simultaneously using the “Enter” method, with no variable selection performed. The logistic regression model was formulated as follows:

(1) $$\begin{array}{ccc} \mathrm{Logit}\left(P\right)=\ln\left(\frac{P}{1-P}\right) & = & \beta_{0}+\beta_{1}*\left[\mathrm{Biomarker}\;1\right]\\ & & +\;\beta_{2}*\left[\mathrm{Biomarker}\;2\right]+\cdot \\ & & +\;\beta_{n}*\left[\mathrm{Biomarker}\;n\right] \end{array}$$

where P is the probability of having cancer, β_0_ is the intercept, and β_1_, β_2_, …, β_n_ are the regression coefficients for the respective biomarkers. The detailed logistic regression equations for each biomarker combination are provided in Supplementary Table 8.

The predicted probability (P) for each subject, derived from the fitted logistic regression model, was defined as the “Combined Diagnostic Index”. This index served as a new, integrated diagnostic variable. The ROC curve for this Combined Diagnostic Index was then constructed, and its AUC, along with the corresponding sensitivity, specificity, and other metrics at its optimal cutoff (determined by the maximum Youden Index), were calculated to evaluate the performance of the biomarker combination.

Spearman's rank correlation (r) was used to assess the correlation between the percentage of platelets with indicated α‐granule patterns and age.

All statistical analyses were performed using SPSS (RRID: SCR_002865) version 26.0. All scientific graphs were generated using GraphPad Prism (RRID: SCR_002798) version 9.3.

## Author contributions

L.C., Ha.D., J.G., Z.H. and Y.‐H.Z. conceived and designed the study. Y.M. developed workflows for sample preparation and advanced imaging. H.D., Z.L., S.Z., L.W., C.Z., H.Z., H.S., N.J., M.H., L.D., Y.B., Cj.Z., Z.Ho., Z.Q., X.W., L.C., Ha.D., J.G. and Z.H. conducted sample and clinical data collection across multiple cohorts, with H.D., L.W., C.Z., H.Z. and Z.H. focusing on gynecological tumors, S.Z., Y.B. and Ha.D. on hepatocellular carcinoma, Z.L., M.H., H.S. and J.G. on prostate cancer, N.J., Cj.Z., L.C. and X.W. on non‐small cell lung cancer, L.D. and Z.Q. on gastric cancer, and Z.Ho. on myeloproliferative neoplasms. Z.L., S.Z., N.J., L.D., Z.Y., Sh.Z., X.H., M.H. and H.S. performed mechanism experiments. H.D., Z.L., S.Z., N.J., Sh.Z. and Y.‐H.Z. analyzed the data and designed figures. Y.M., H.D., Z.L., S.Z., N.J. and Y.‐H.Z. wrote the manuscript. X.‐Y.H. provided essential operational support. J.X., S.Z., J. Y., Q. L. provided critical comments. All authors reviewed and edited the paper. Y.‐H.Z. supervised the project.

## Conflicts of Interest

The authors declare no conflict of interest.

## Supporting information




**Supporting File**: advs75094‐sup‐0001‐SuppMat.docx.

## Data Availability

The data that support the findings of this study are available from the corresponding author upon reasonable request.

## References

[1] P. Song , L. R. Wu , Y. H. Yan , et al., “Limitations and Opportunities of Technologies for the Analysis of Cell‐free DNA in Cancer Diagnostics,” Nature Biomedical Engineering 6 (2022): 232–245. 10.1038/s41551-021-00837-3 PMC9336539 35102279

[2] S. Y. Shen , R. Singhania , G. Fehringer , et al., “Sensitive Tumour Detection and Classification Using Plasma Cell‐free DNA Methylomes,” Nature 563 (2018): 579–583. 30429608 10.1038/s41586-018-0703-0

[3] M. Ignatiadis , G. W. Sledge , and S. S. Jeffrey , “Liquid biopsy enters the clinic — implementation issues and future challenges,” Nature Reviews Clinical Oncology 18 (2021): 297–312. 10.1038/s41571-020-00457-x 33473219

[4] S. M. Batool , A. Yekula , P. Khanna , et al., “The Liquid Biopsy Consortium: Challenges and Opportunities for Early Cancer Detection and Monitoring,” Cell Reports Medicine 4 (2023): 101198. 37716353 10.1016/j.xcrm.2023.101198 PMC10591039

[5] A. M. Aravanis , M. Lee , and R. D. Klausner , “Next‐generation Sequencing of Circulating Tumor DNA for Early Cancer Detection,” Cell 168 (2017): 571–574. 28187279 10.1016/j.cell.2017.01.030

[6] J. D. Cohen , L. Li , Y. Wang , et al., “Detection and Localization of Surgically Resectable Cancers With a Multi‐analyte Blood Test,” Science 359 (2018): 926–930. 29348365 10.1126/science.aar3247 PMC6080308

[7] J. Phallen , M. Sausen , V. Adleff , et al., “Direct Detection of Early‐stage Cancers Using Circulating Tumor DNA,” Science Translational Medicine 9 (2017): aan2415. 10.1126/scitranslmed.aan2415 PMC6714979 28814544

[8] C. Martin‐Alonso , S. Tabrizi , K. Xiong , et al., “Priming Agents Transiently Reduce the Clearance of Cell‐free DNA to Improve Liquid Biopsies,” Science 383 (2024): adf2341. 10.1126/science.adf2341 PMC11529396 38236959

[9] W. Li , J.‐B. Liu , L.‐K. Hou , et al., “Liquid Biopsy in Lung Cancer: Significance in Diagnostics, Prediction, and Treatment Monitoring,” Molecular Cancer 21 (2022): 25. 35057806 10.1186/s12943-022-01505-z PMC8772097

[10] M. Chen , L. Hou , L. Hu , et al., “Platelet Detection as a New Liquid Biopsy Tool for human Cancers,” Frontiers in Oncology 12 (2022): 983724. 36185270 10.3389/fonc.2022.983724 PMC9515491

[11] S. Li , Z. Lu , S. Wu , et al., “The Dynamic Role of Platelets in Cancer Progression and Their Therapeutic Implications,” Nature Reviews Cancer 24 (2024): 72–87. 38040850 10.1038/s41568-023-00639-6

[12] S. D'Ambrosi , R. J. Nilsson , and T. Wurdinger , “Platelets and Tumor‐associated RNA Transfer,” Blood 137 (2021): 3181–3191. 33940602 10.1182/blood.2019003978 PMC8351881

[13] H. G. Roweth and E. M. Battinelli , “Lessons to Learn From Tumor‐educated Platelets,” Blood 137 (2021): 3174–3180. 33940594 10.1182/blood.2019003976 PMC8351883

[14] P. E. J. van der Meijden and J. W. M. Heemskerk , “Platelet Biology and Functions: New Concepts and Clinical Perspectives,” Nature Reviews Cardiology 16 (2019): 166–179. 30429532 10.1038/s41569-018-0110-0

[15] P. Xu , H. Deng , Z. Hong , et al., “Superresolution Fluorescence Microscopy of Platelet Subcellular Structures as a Potential Tumor Liquid Biopsy,” Small Methods 7 (2023): 2300445. 10.1002/smtd.202300445 37349902

[16] R. Wang , R. L. Stone , J. T. Kaelber , et al., “Electron Cryotomography Reveals Ultrastructure Alterations in Platelets From Patients With Ovarian Cancer,” Proceedings of the National Academy of Sciences 112 (2015): 14266–14271. 10.1073/pnas.1518628112 PMC4655568 26578771

[17] P. K. Chaudhary , S. Kim , and S. Kim , “An Insight Into Recent Advances on Platelet Function in Health and Disease,” International Journal of Molecular Sciences 23 (2022): 6022. 35682700 10.3390/ijms23116022 PMC9181192

[18] H. Deng , Y. Ma , and Y.‐H. Zhang , “Super‐resolution Microscopy: Shedding New Light on Blood Cell Imaging,” Journal of Innovative Optical Health Sciences 18 (2025): 2430006.

[19] J. Liu , Y. Y. Tan , W. Zheng , Y. Wang , L. A. Ju , and Q. P. Su , “Nanoscale Insights Into Hematology: Super‐resolved Imaging on Blood Cell Structure, Function, and Pathology,” Journal of Nanobiotechnology 22 (2024): 363. 38910248 10.1186/s12951-024-02605-2 PMC11194919

[20] I. De Simone , C. C. F. M. J. Baaten , J. M. Gibbins , et al., “Repeated Platelet Activation and the Potential of Previously Activated Platelets to Contribute to Thrombus Formation,” Journal of Thrombosis and Haemostasis 21 (2023): 1289–1306. 36754678 10.1016/j.jtha.2023.01.006

[21] J. Chung , D. Jeong , G.‐H. Kim , et al., “Super‐resolution Imaging of Platelet‐activation Process and Its Quantitative Analysis,” Scientific Reports 11 (2021): 10511. 34006947 10.1038/s41598-021-89799-9 PMC8131365

[22] J. J. Fenton , M. S. Weyrich , S. Durbin , et al., “Prostate‐specific Antigen–based Screening for Prostate Cancer,” Jama 319 (2018): 1914–1931. 29801018 10.1001/jama.2018.3712

[23] F. Crocetto , M. Musone , S. Chianese , et al., “Blood and Urine‐based Biomarkers in Prostate Cancer: Current Advances, Clinical Applications, and Future Directions,” The Journal of Liquid Biopsy 9 (2025): 100305. 40606769 10.1016/j.jlb.2025.100305 PMC12221370

[24] F. Crocetto , G. Russo , E. Di Zazzo , et al., “Liquid Biopsy in Prostate Cancer Management—Current Challenges and Future Perspectives,” Cancers 14 (2022): 3272. 35805043 10.3390/cancers14133272 PMC9265840

[25] T. A. Stamey , N. Yang , A. R. Hay , J. E. McNeal , F. S. Freiha , and E. Redwine , “Prostate‐specific Antigen as a Serum Marker for Adenocarcinoma of the Prostate,” New England Journal of Medicine 317 (1987): 909–916. 2442609 10.1056/NEJM198710083171501

[26] S. Roy , M. Kanda , S. Nomura , et al., “Diagnostic Efficacy of Circular RNAs as Noninvasive, Liquid Biopsy Biomarkers for Early Detection of Gastric Cancer,” Molecular Cancer 21 (2022): 42. 35139874 10.1186/s12943-022-01527-7 PMC8826675

[27] R. Chen , L.‐Q. Zhou , X.‐B. Cai , et al., “Percent free prostate‐specific antigen is effective to predict prostate biopsy outcome in Chinese men With prostate‐specific antigen Between 10.1 and 20.0 ng ml −1,” Asian Journal of Andrology 17 (2015): 1017–1021. 25926603 10.4103/1008-682X.150846 PMC4814972

[28] R. Chen , D. D. Sjoberg , Y. Huang , et al., “Prostate Specific Antigen and Prostate Cancer in Chinese Men Undergoing Initial Prostate Biopsies Compared With Western Cohorts,” Journal of Urology 197 (2017): 90–96. 27593477 10.1016/j.juro.2016.08.103 PMC5503119

[29] R. Chen , L. Xie , X. Cai , et al., “Percent free prostate‐specific antigen for prostate cancer diagnosis in Chinese men With a PSA of 4.0–10.0 ng/mL: Results From the Chinese Prostate Cancer Consortium,” Asian Journal of Urology 2 (2015): 107–113. 29264128 10.1016/j.ajur.2015.04.022 PMC5730736

[30] M. Ferro , F. Crocetto , E. La Civita , et al., “Serum (‐2)proPSA/freePSAratio, (‐2)proPSA/freePSA density, prostate health index, and prostate health index density as clues to reveal postoperative clinically significant prostate cancer in men With prostate‐specific antigen 2–10 ng/mL,” The Prostate 84 (2024): 1157–1164. 38798011 10.1002/pros.24752

[31] T. Nordström , A. Vickers , M. Assel , H. Lilja , H. Grönberg , and M. Eklund , “Comparison Between the Four‐kallikrein Panel and Prostate Health Index for Predicting Prostate Cancer,” European Urology 68 (2015): 139–146. 25151013 10.1016/j.eururo.2014.08.010 PMC4503229

[32] M. Kim , C. Chen , P. Wang , et al., “Detection of Ovarian Cancer via the Spectral Fingerprinting of Quantum‐defect‐modified Carbon Nanotubes in Serum by Machine Learning,” Nature Biomedical Engineering 6 (2022): 267–275. 10.1038/s41551-022-00860-y PMC9108893 35301449

[33] I. J. Jacobs , U. Menon , A. Ryan , et al., “Ovarian Cancer Screening and Mortality in the UK Collaborative Trial of Ovarian Cancer Screening (UKCTOCS): A Randomised Controlled Trial,” The Lancet 387 (2016): 945–956. 10.1016/S0140-6736(15)01224-6 PMC4779792 26707054

[34] U. Menon , A. Gentry‐Maharaj , M. Burnell , et al., “Ovarian Cancer Population Screening and Mortality After Long‐term Follow‐up in the UK Collaborative Trial of Ovarian Cancer Screening (UKCTOCS): A Randomised Controlled Trial,” The Lancet 397 (2021): 2182–2193. 10.1016/S0140-6736(21)00731-5 PMC8192829 33991479

[35] U. Menon , M. Griffin , and A. Gentry‐Maharaj , “Ovarian Cancer Screening—Current Status, Future Directions,” Gynecologic Oncology 132 (2014): 490–495. 24316306 10.1016/j.ygyno.2013.11.030 PMC3991859

[36] R. Zhang , M. K. Y. Siu , H. Y. S. Ngan , and K. K. L. Chan , “Molecular Biomarkers for the Early Detection of Ovarian Cancer,” International Journal of Molecular Sciences 23 (2022): 12041. 36233339 10.3390/ijms231912041 PMC9569881

[37] W. Froyman , C. Landolfo , B. De Cock , et al., “Risk of Complications in Patients With Conservatively Managed Ovarian Tumours (IOTA5): A 2‐year Interim Analysis of a Multicentre, Prospective, Cohort Study,” The Lancet Oncology 20 (2019): 448–458. 30737137 10.1016/S1470-2045(18)30837-4

[38] Y. Gao , S. Zeng , X. Xu , et al., “Deep Learning‐enabled Pelvic Ultrasound Images for Accurate Diagnosis of Ovarian Cancer in China: A Retrospective, Multicentre, Diagnostic Study,” The Lancet Digital Health 4 (2022): e179–e187. 35216752 10.1016/S2589-7500(21)00278-8

[39] Y. Gao , C. J. Liu , H. Y. Li , et al., “Platelet RNA Enables Accurate Detection of Ovarian Cancer: An Intercontinental, Biomarker Identification Study,” Protein Cell 14 (2023): 579–590. 36905391 10.1093/procel/pwac056 PMC10246718

[40] P. Giampaolino , V. Foreste , L. Della Corte , C. Di Filippo , G. Iorio , and G. Bifulco , “Role of Biomarkers for Early Detection of Ovarian Cancer Recurrence,” Gland Surgery 9 (2020): 1102–1111. 32953625 10.21037/gs-20-544 PMC7475347

[41] J. Y. Hou , J. S. Chapman , E. Kalashnikova , et al., “Circulating Tumor DNA Monitoring for Early Recurrence Detection in Epithelial Ovarian Cancer,” Gynecologic Oncology 167 (2022): 334–341. 36117009 10.1016/j.ygyno.2022.09.004

[42] S. G. J. G. In 't Veld , M. Arkani , E. Post , et al., “Detection and Localization of Early‐ and Late‐stage Cancers Using Platelet RNA,” Cancer Cell 40 (2022): 999–1009.e6. 36055228 10.1016/j.ccell.2022.08.006

[43] K. Pastuszak , A. Supernat , M. G. Best , et al., “imPlatelet classifier: Image‐converted RNA biomarker profiles enable blood‐based cancer diagnostics,” Molecular Oncology 15 (2021): 2688–2701. 34013585 10.1002/1878-0261.13014 PMC8486571

[44] L. Murphy , J. Inchauspé , G. Valenzano , et al., “Platelets sequester extracellular DNA, capturing tumor‐derived and free fetal DNA,” Science 389 (2025): adp3971. 10.1126/science.adp3971 PMC7618233 40811534

[45] L. Oliveira‐Ferrer , K. Milde‐Langosch , K. Eylmann , et al., “Mechanisms of Tumor‐Lymphatic Interactions in Invasive Breast and Prostate Carcinoma,” International Journal of Molecular Sciences 21 (2020): 602. 31963450 10.3390/ijms21020602 PMC7013901

[46] T. Kress , C. Barris , S. Kennard , and E. De Chantemèle , “Novel Experimental Approach to Investigate Immune Control of Vascular Function: Co‐culture of Murine Aortas with T Lymphocytes or Macrophages,” BIO‐PROTOCOL 15 (2025): 5440. 10.21769/BioProtoc.5440 PMC12423277 40948901

[47] T. Dudiki , M. Veleeparambil , I. Zhevlakova , et al., “Mechanism of Tumor‐platelet Communications in Cancer,” Circulation Research 132 (2023): 1447–1461. 37144446 10.1161/CIRCRESAHA.122.321861 PMC10213120

[48] L. Zhou , D. Wu , Y. Zhou , et al., “Tumor Cell‐released Kynurenine Biases MEP Differentiation Into Megakaryocytes in Individuals With Cancer by Activating AhR–RUNX1,” Nature Immunology 24 (2023): 2042–2052. 37919525 10.1038/s41590-023-01662-3 PMC10681900

[49] M. Lomnytska , R. Pinto , S. Becker , et al., “Platelet Protein Biomarker Panel for Ovarian Cancer Diagnosis,” Biomarker Research 6 (2018): 2. 29344361 10.1186/s40364-018-0118-y PMC5767003

[50] M. G. Best , N. Sol , I. Kooi , et al., “RNA‐seq of Tumor‐educated Platelets Enables Blood‐based Pan‐cancer, Multiclass, and Molecular Pathway Cancer Diagnostics,” Cancer Cell 28 (2015): 666–676. 26525104 10.1016/j.ccell.2015.09.018 PMC4644263

[51] F. Ren , Q. Fei , K. Qiu , Y. Zhang , H. Zhang , and L. Sun , “Liquid Biopsy Techniques and Lung Cancer: Diagnosis, Monitoring and Evaluation,” Journal of Experimental & Clinical Cancer Research 43 (2024): 96. 38561776 10.1186/s13046-024-03026-7 PMC10985944

[52] G. Lippi , “Genetic and Nongenetic Determinants of Mean Platelet Volume,” Blood 127 (2016): 179–180. 26769773 10.1182/blood-2015-11-679852

[53] L. Zhao , C. D. Kroenke , J. Song , D. Piwnica‐Worms , J. J. H. Ackerman , and J. J. Neil , “Intracellular water‐specific MR of microbead‐adherent cells: The HeLa cell intracellular water exchange lifetime,” NMR in Biomedicine 21 (2008): 159–164. 17461436 10.1002/nbm.1173 PMC2700831

[54] L. C. E. Giudice , “Endometriosis,” New England Journal of Medicine 362 (2010): 2389–2398. 20573927 10.1056/NEJMcp1000274 PMC3108065

[55] H. Xiang , Y. Xiao , F. Li , et al., “Development and Validation of an Interpretable Model Integrating Multimodal Information for Improving Ovarian Cancer Diagnosis,” Nature Communications 15 (2024): 2681. 10.1038/s41467-024-46700-2 PMC10973484 38538600

