# Supplementary material for: Large‐Scale Quantitative Morphometry of Platelet α‐Granules via SIM Super‐Resolution Microscopy for Cancer Liquid Biopsy
Source: Adv Sci (Weinh). 2026 Apr 10;13(34):e75094. doi: 10.1002/advs.75094 (PMC13285143; doi:10.1002/advs.75094)
Supplement: Supplementary file 1 — Supporting File: advs75094‐sup‐0001‐SuppMat.docx. [file ADVS-13-e75094-s001.docx]

**Supplemental materials**

**Large-scale quantitative morphometry of platelet α-granules**

**via SIM super-resolution microscopy for cancer liquid biopsy**

Yan Ma, Huan Deng, Zelin Liu, Simei Zhong, and Liangliang Wang contributed equally to this work and shared the first authorship.

Yan Ma^1,2,13^, Huan Deng^1,2,13^, Zelin Liu^1,13^, Simei Zhong^1,13^, Liangliang Wang^3,13^, Mu He^4^, Ning Jing^1^, Lihua Dai^2^, Yuzhen Basang^5^, Xiao-Yu Hu^2^, Changyi Zhang^3^, Huimin Zeng^3^, Haoren Shao^4^, Zhe Yang^1^, Shanshan Zhao^1^, Xiaohai Hu^1^, Chuanjie Zhang^6^, Xiaowei Wu^6^, Jing Xu^2^, Shaoqun Zeng^1^, Jing Yuan^1^, Qiang Li^1^, Ziwei Qu^7^, Zhenya Hong^8^, Lingjuan Chen^9,10✉^, Hanhua Dong^5,11✉^, Jia Guo^4✉^, Zhiqiang Han^3,12✉^, Yu-Hui Zhang^1✉^

**Extended Data Figure 1 |** Representative SIM images of α-granule distributions in in healthy donor (HD), Non-malignant disease patients (NMD), and patients with nine cancers. Scale bar, 5 μm.


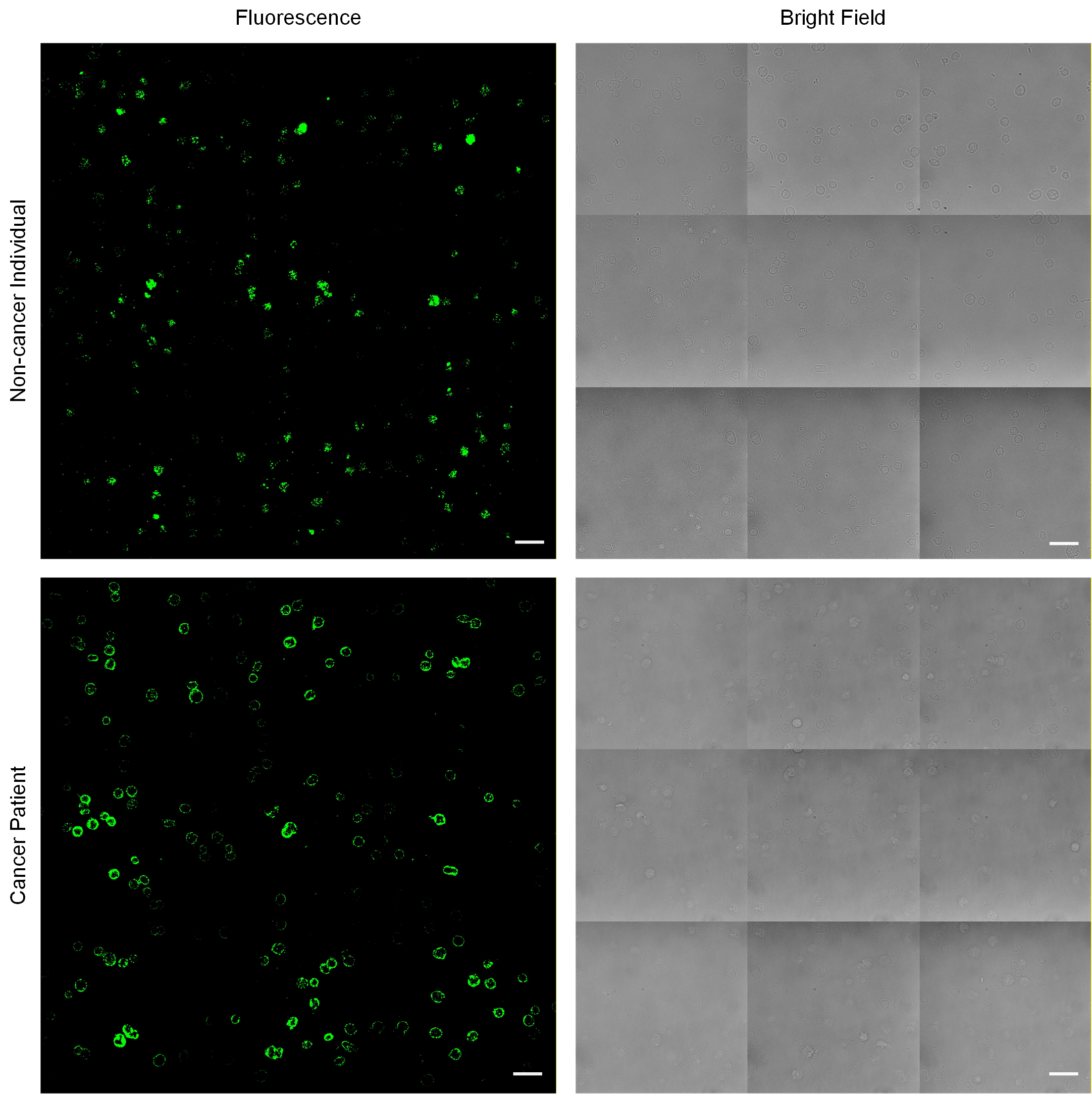


**Extended Data Figure 2** | 3×3 Tile-scan SIM images of platelet α-granules from a non-cancer individual and a cancer patient: the left panel shows the fluorescence channel, and the right panel shows the corresponding bright-field image. Scale bar, 10 μm.


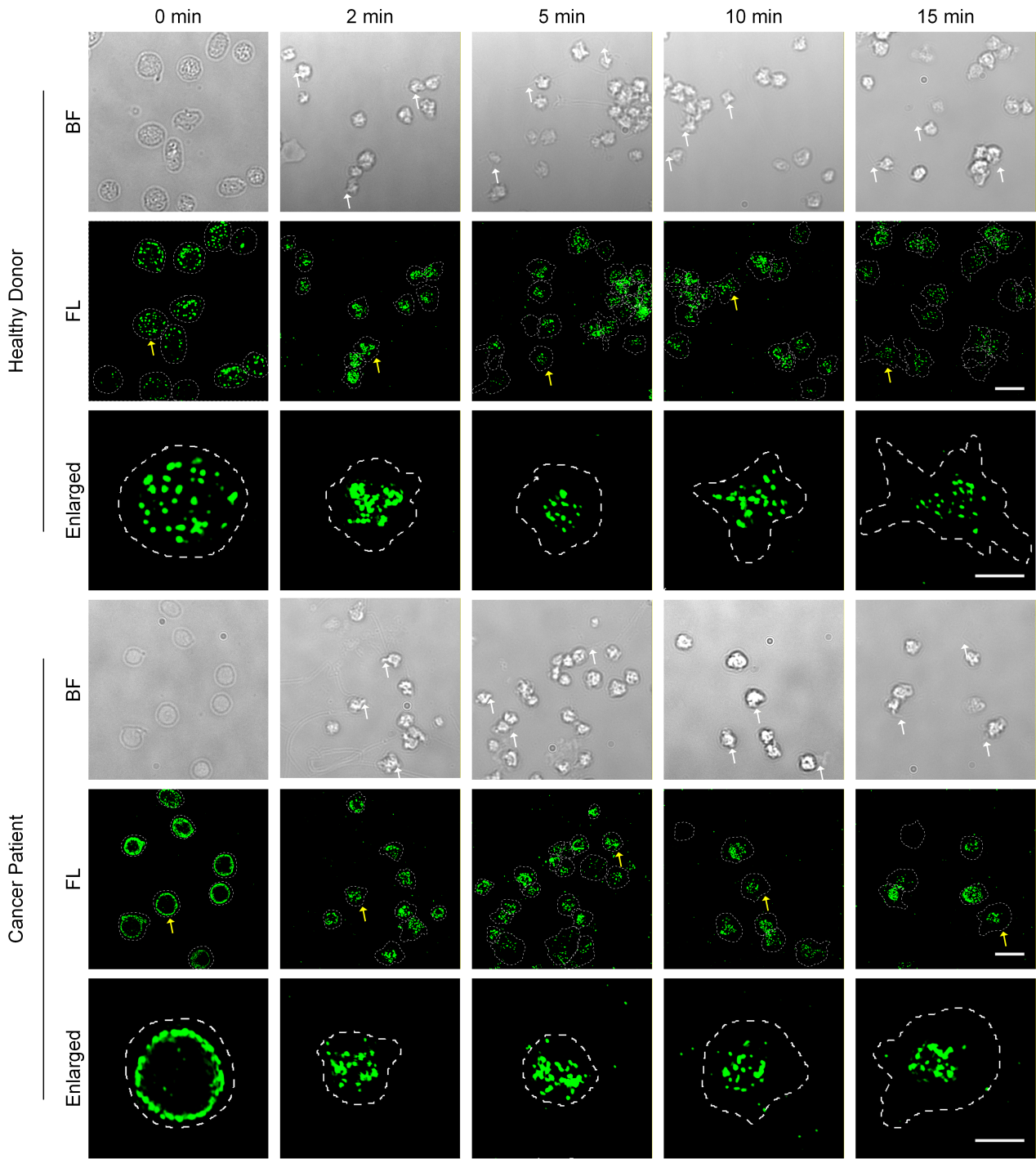


**Extended Data Figure 3 |** Representative bright-field images of platelets and SIM images of α-granules from healthy donors (top, n = 3) and cancer patients (bottom, n = 3) at 0, 2, 5, 10, and 15 minutes after 2.5 U/mL thrombin stimulation. White arrows indicate platelet filopodia; yellow arrows indicate the platelets shown in the enlarged views; dashed outlines delineate platelet boundaries. Scale bars, 5 μm for the original images and 2.5 μm for the enlarged views. BF, bright-field; FL, fluorescence.


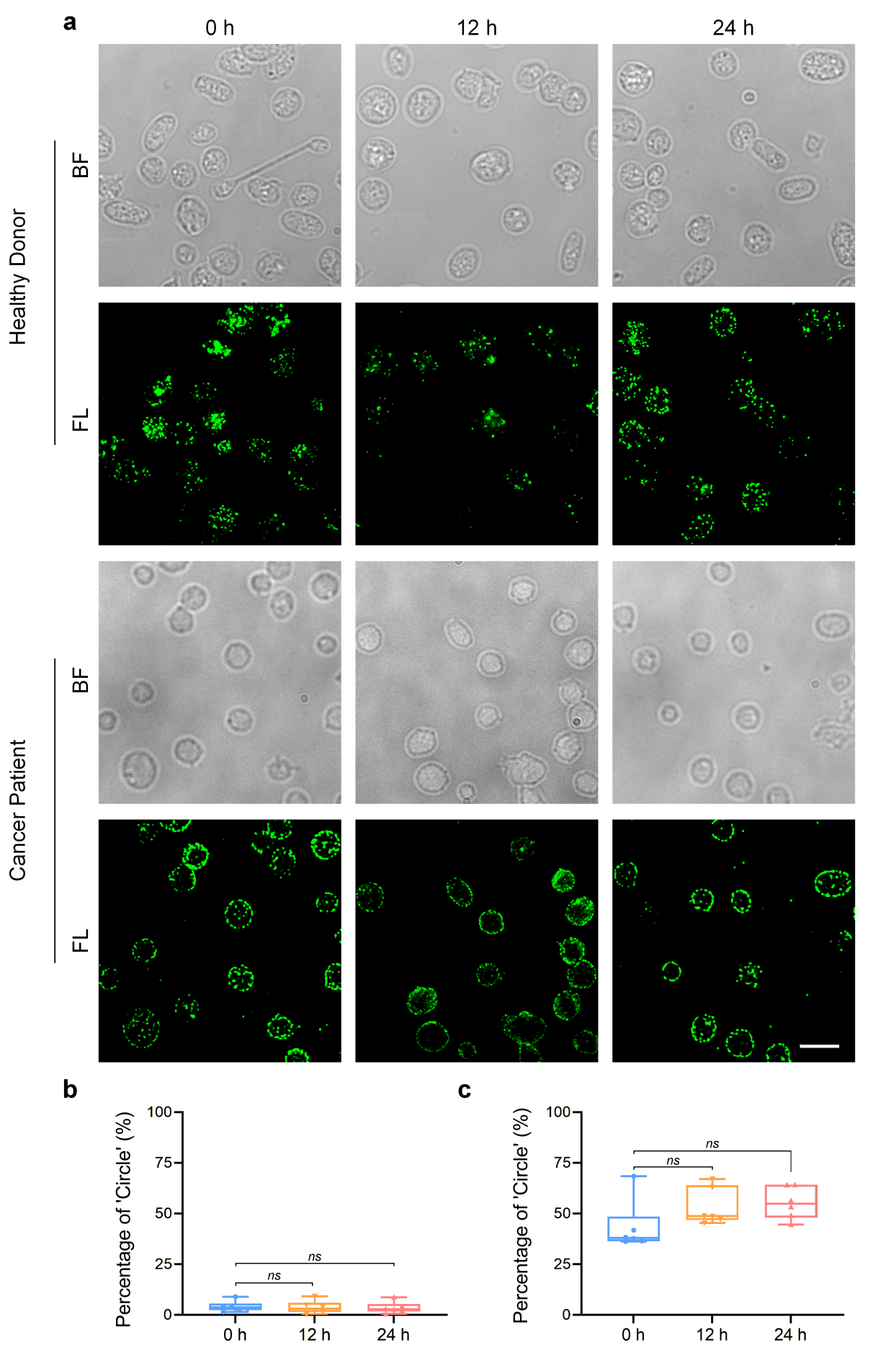


**Extended Data Figure 4** | **a,** Representative bright-field images of platelets and SIM images of α-granules from healthy donors (top) and cancer patients (bottom) after whole-blood storage at room temperature for 0, 12, and 24 hours. **b, c,** Comparison of the ‘Circle’ pattern percentage in platelets from healthy donors (n = 6) and cancer patients (n = 6) after whole-blood storage at room temperature for 0, 12, and 24 hours. Data are presented as mean ± SD (n = 6; the one-way ANOVA with Dunnett’s post-test for multiple comparisons for three groups). The ‘Circle’ percentage remained unchanged across storage durations. Scale bar, 5 μm. *ns*: non-significant difference.

**Extended Data Figure 5** | **a,** Percentages of the ‘Circle’ pattern in HD, patients with cirrhosis, and patients with HCC. The percentage of ‘Circle’ pattern was significantly higher in HCC than in the other two groups (Kruskal-Wallis test with Bonferroni correction). **b,** Percentages of the ‘Circle’ pattern in HCC without cirrhosis (cirrhosis (-)) and HCC with underlying cirrhosis (cirrhosis (+)). The values were statistically indistinguishable (Mann–Whitney U test).


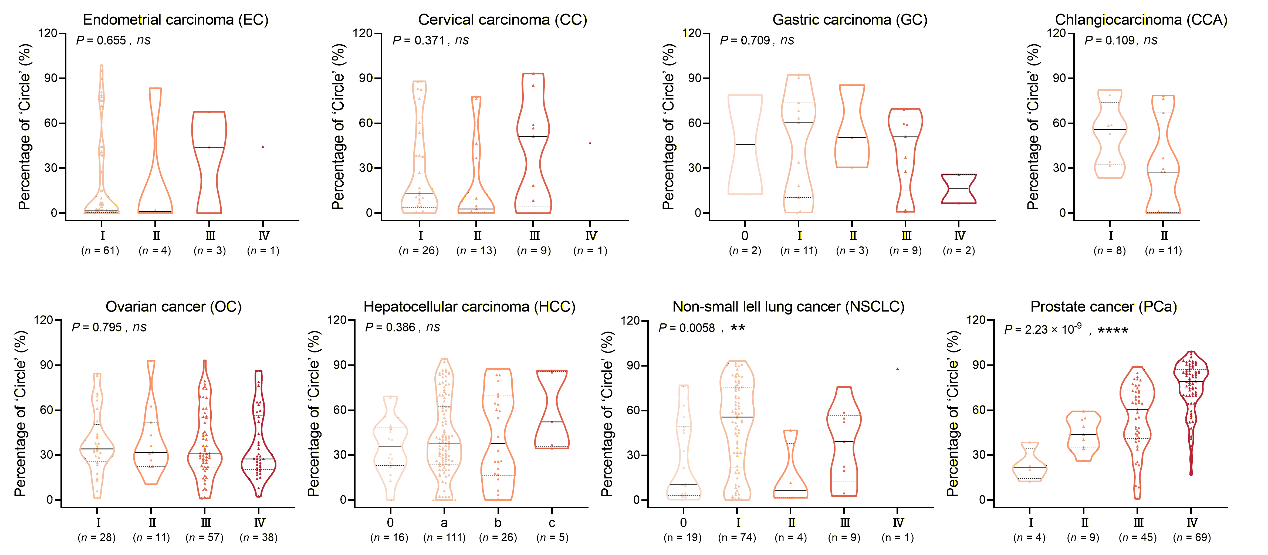


**Extended Data Figure 6** | Comparison of ‘Circle’ percentage across different clinical stages in eight cancer types. Violin plots display the distribution of ‘Circle’ percentages for each cancer type, with the solid line representing the median and dashed lines the interquartile range (IQR). Statistical significance across stages was assessed by the Kruskal-Wallis test (*ns*, *P* ≥ 0.05; ***P* < 0.01; *****P* < 0.0001). Staging systems: OC, CC, and EC were staged according to FIGO (International Federation of Gynecology and Obstetrics); NSCLC, PCa, and GC according to AJCC (American Joint Committee on Cancer); and HCC according to BCLC (Barcelona Clinic Liver Cancer).

**Extended Data Figure 7 | a,** Distribution of platelet α-granule patterns in BPH and PCa patients. ‘Circle’ pattern was increased in PCa patients (Mann-Whitney *U* test). **b, c,** Sensitivity (left) and specificity (right) of PAID, PSA, and PAID combined with PSA for distinguishing PCa from BPH in and validation cohort A (b) and cohort B (c). PSA was classified here according to clinical criteria: values > 10 ng/mL were considered indicative of cancer, whereas values < 4 ng/mL were considered non-cancer. PAID, and PAID combined with PSA, demonstrated improved diagnostic performance (higher sensitivity and specificity compared to PSA alone. **d,** Distribution of platelet α-granule patterns in BPH and PCa patients with PSA levels of 4-10 ng/mL. ‘Circle’ pattern was increased in PCa patients (Mann-Whitney *U* test). **e,** Illustrative clinical case: A patient with prostate adenocarcinoma (acinar type) diagnosed by biopsy and correctly identified by PAID, but missed by PSA and mpMRI. Scale bar, 2 μm.


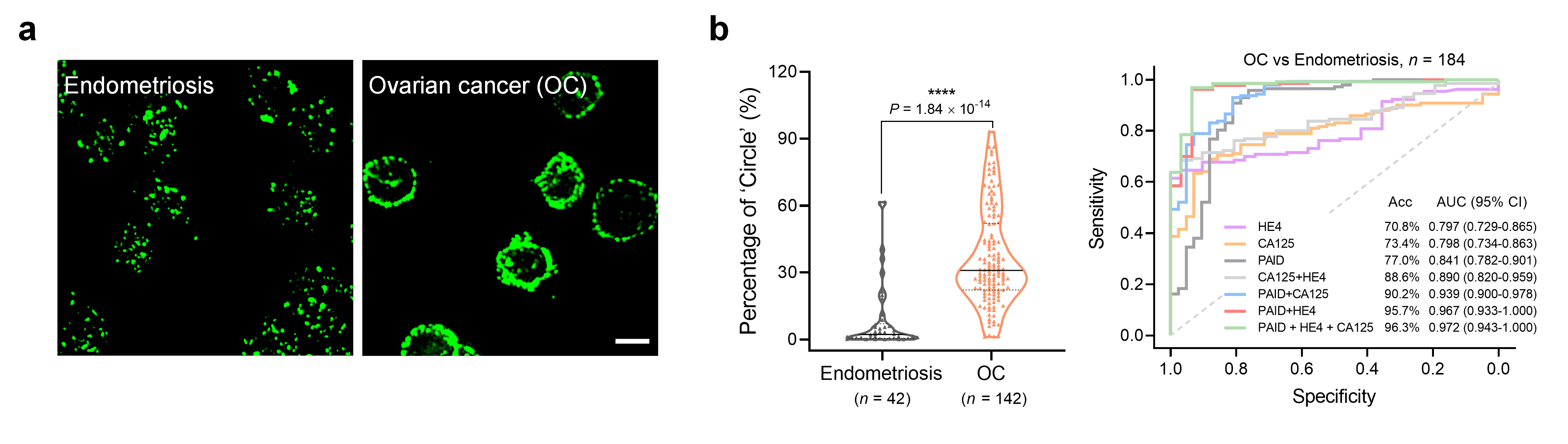


**Extended Data Figure 8 |** **a,** Representative SIM images of α-granule distributions in endometriosis (left, ‘N ≤ 30’ pattern) and ovarian cancer (OC, right, ‘Circle’ pattern). Scale bars, 2 μm. **b,** ‘Circle’ percentage in endometriosis and OC. ‘Circle’ pattern was significantly increased in OC (Mann-Whitney *U* test). ROC curve evaluates the diagnostic performance of PAID, CA125, HE4, and their combination for discriminating OC from endometriosis. PAID combined with HE4 and all biomarkers combined showed improved diagnostic performance (higher AUCs) compared to others.

**Extended Data Figure 9 |** **Illustrative clinical examples misdiagnosed as ovarian cancer (OC) or benign adnexal mass cases (BAM).** **a,** A patient with OC (borderline serous tumor) diagnosed by biopsy and correctly identified by PAID, but initially misdiagnosed as BAM based on CA125 and TVS. **b,** A patient with endometriosis diagnosed by biopsy and correctly identified by PAID, but initially misdiagnosed as OC based on CA125 and CT. **c,** A patient with tubal mesosalpinx cyst diagnosed by biopsy and correctly identified by PAID, but initially misdiagnosed as OC based on CA125 levels and TVS imaging. Scale bar, 2 μm.

**Extended Data Figure 10 |** **Illustrative clinical case:** Longitudinal monitoring of two patients without recurrence using PAID, CA125, and HE4 at various time points before surgery, during adjuvant chemotherapy, and during follow-up. Scale bar, 2 μm.

**Extended Data Figure 11 |** **Optimization of platelet and cancer cell co-incubation conditions.** **a,** Distribution of α-granule patterns in platelet incubated under standard storage conditions (ACDT, 22°C) and in tumor cell culture media (RPMI 1640, 37°C). Statistical analysis revealed no significant difference (the Student’s *t*-test for two groups). **b,** **c,** Representative SIM images (b) and ‘Circle’ percentage (c) of α-granule after co-incubation with cancer cells in a tumor microenvironment-mimicking condition for 0, 6, 12, and 18 hours. ‘Circle’ percentage was significantly increased after 12 hours co-incubation with PC-3 and SK-OV-3 cells. Scale bar, 5 μm.


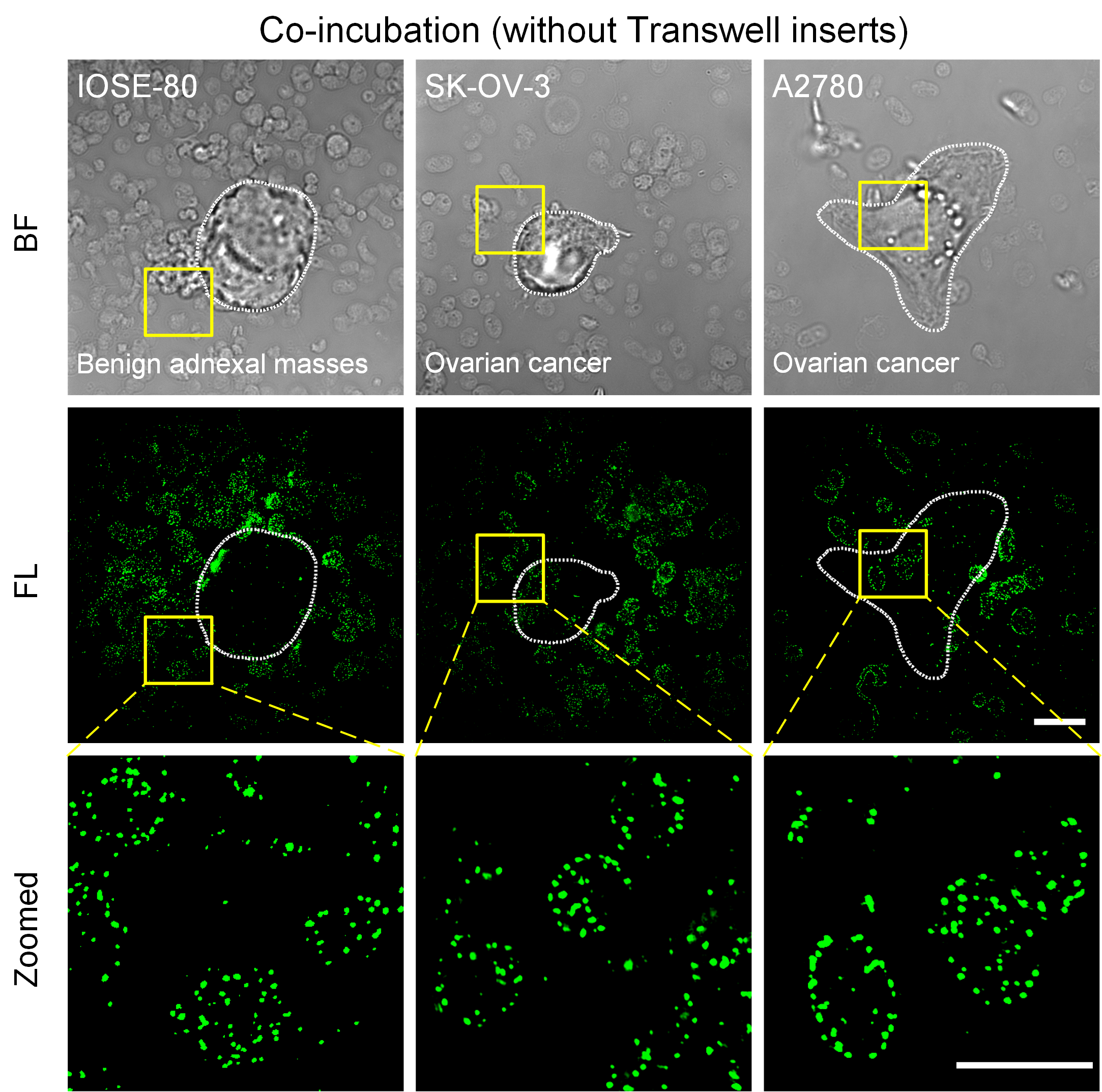


**Extended Data Figure 12 | Co-incubation (without Transwell inserts):** Representative bright-field (BF, top) and SIM images (FL, middle) of platelets after 12 h contact with benign (IOSE-80) and cancer (SK-OV-3 and A2780) cells. Zoomed insets (bottom) highlight the boxed regions. The white dotted line represents the boundary of a benign or cancer cell. ‘Circle’ pattern was significantly increased in platelets adjacent to both benign (IOSE-80) and cancer (SK-OV-3 and A2780) cells. Scale bar, 5 μm.


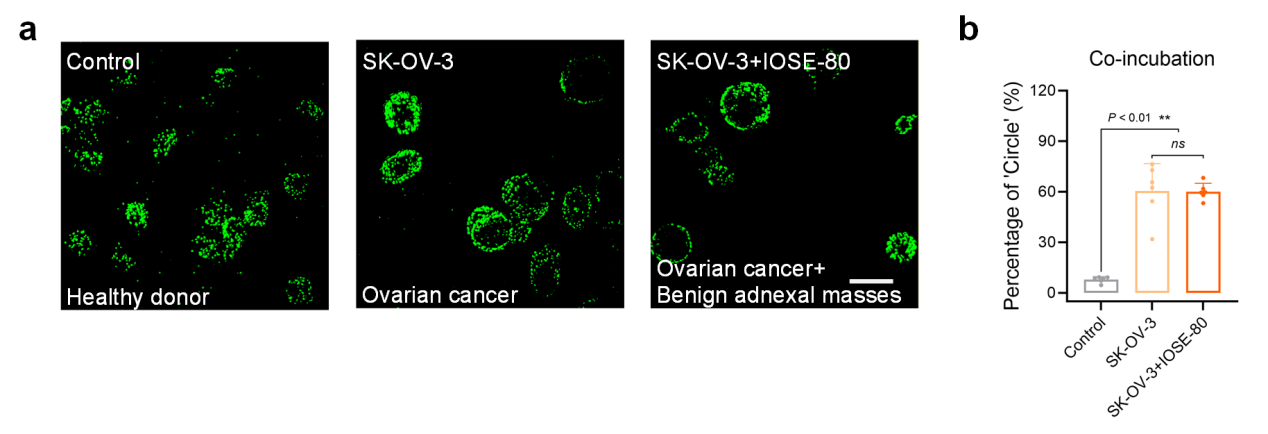
**Extended Data Figure 13 | a, b,** Representative SIM images (a) and percentages of the ‘Circle’ pattern (b) in platelet α-granule distributions under a cell-free conditions, after 12 h co-incubation with SK-OV-3, or after sequential incubation with SK-OV-3 for 12 h and followed by IOSE80 for 12 h. Data are presented as mean ± SD (n = 6; the one-way ANOVA with Dunnett’s post-test for multiple comparisons). Platelets pre-incubated with tumor cells maintained an elevated ‘Circle’ proportion, and subsequent co-incubation with the benign cell line did not reduce this proportion. Scale bar, 5 μm.


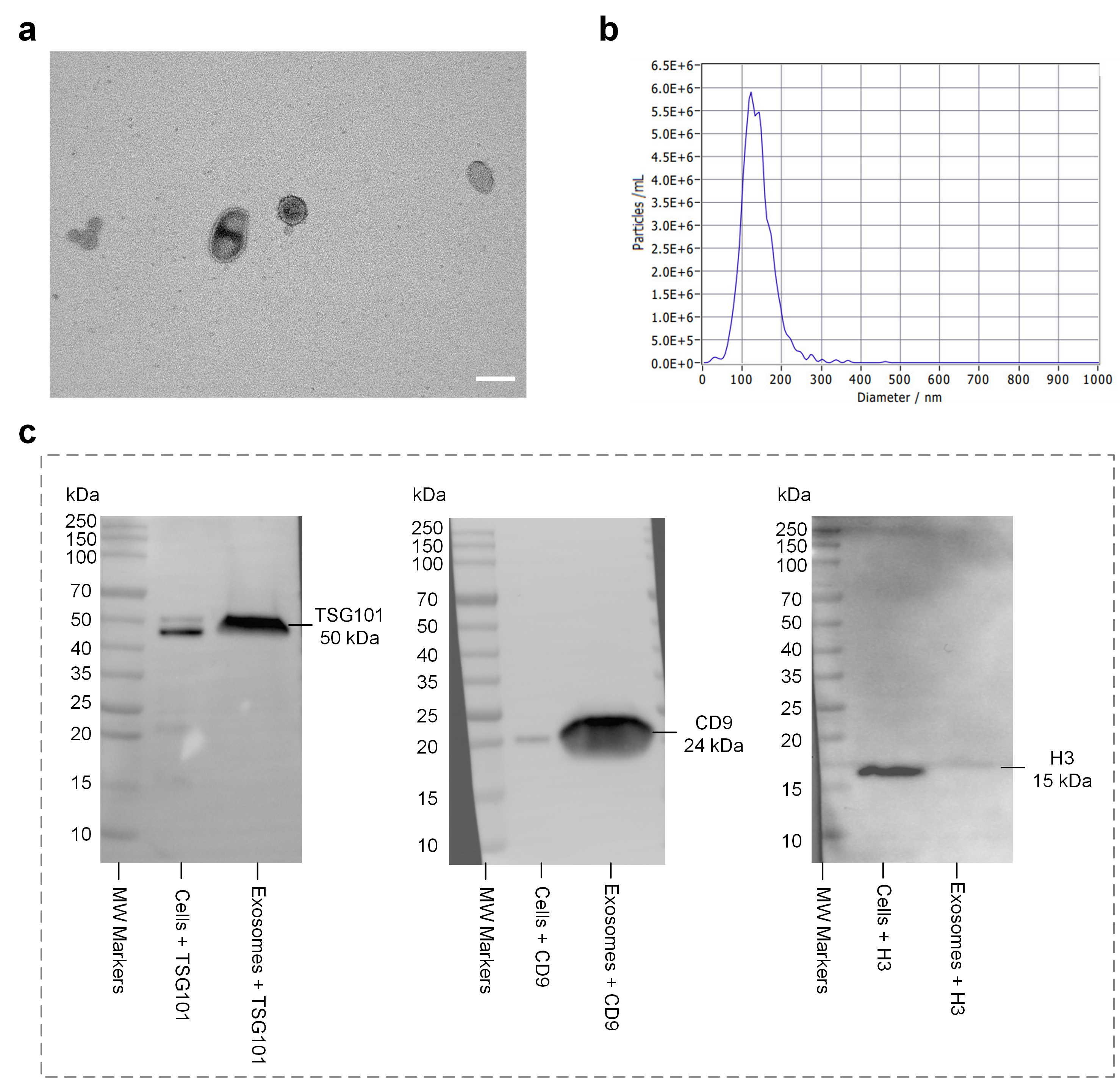


**Extended Data Figure 14 |** **Characterization of tumor-derived exosomes.** **a,** Representative transmission electron microscopy (TEM) image of exosomes showing the characteristic cup-shaped morphology. Scale bar, 100 nm. **b,** Nanoparticle tracking analysis (NTA) quantification showing the size distribution of exosomes, with a mode diameter of approximately 120 nm. **c,** Western blot analysis confirming the presence of exosome markers CD9 and TSG101, while histone H3 was undetectable.


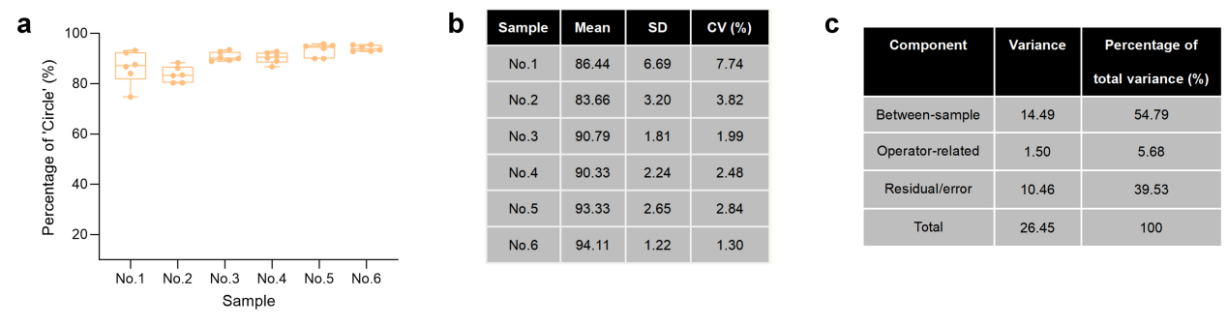


**Extended Data Figure 15** **| Inter-operator reproducibility of ‘Circle’ pattern quantification.** **a,** Percentages of ‘Circle’ pattern independently quantified by six operators from different centers for six samples (No.1-6). **b,** Mean, SD, and inter-operator CV of ‘Circle’ percentage for each sample (n = six operators). CV = SD / mean × 100% (SD: standard deviation; CV: coefficient of variation). **c,** Variance components estimated from a two-way random-effects mixed model (Y_ij_ = μ + u_sample_,_i_ + u_operator_,_j_ + ε_ij_, Y_ij_: the measurement recorded for sample i by operator j; μ: overall mean; u*_sample,i_*, u*_operator,j_*, and *ε_ij_*: random effects of sample, operator, and residual error, respectively) and expressed as percentages of the total variance.

**Extended Data Table 1 | Patient demographics and clinical information.**


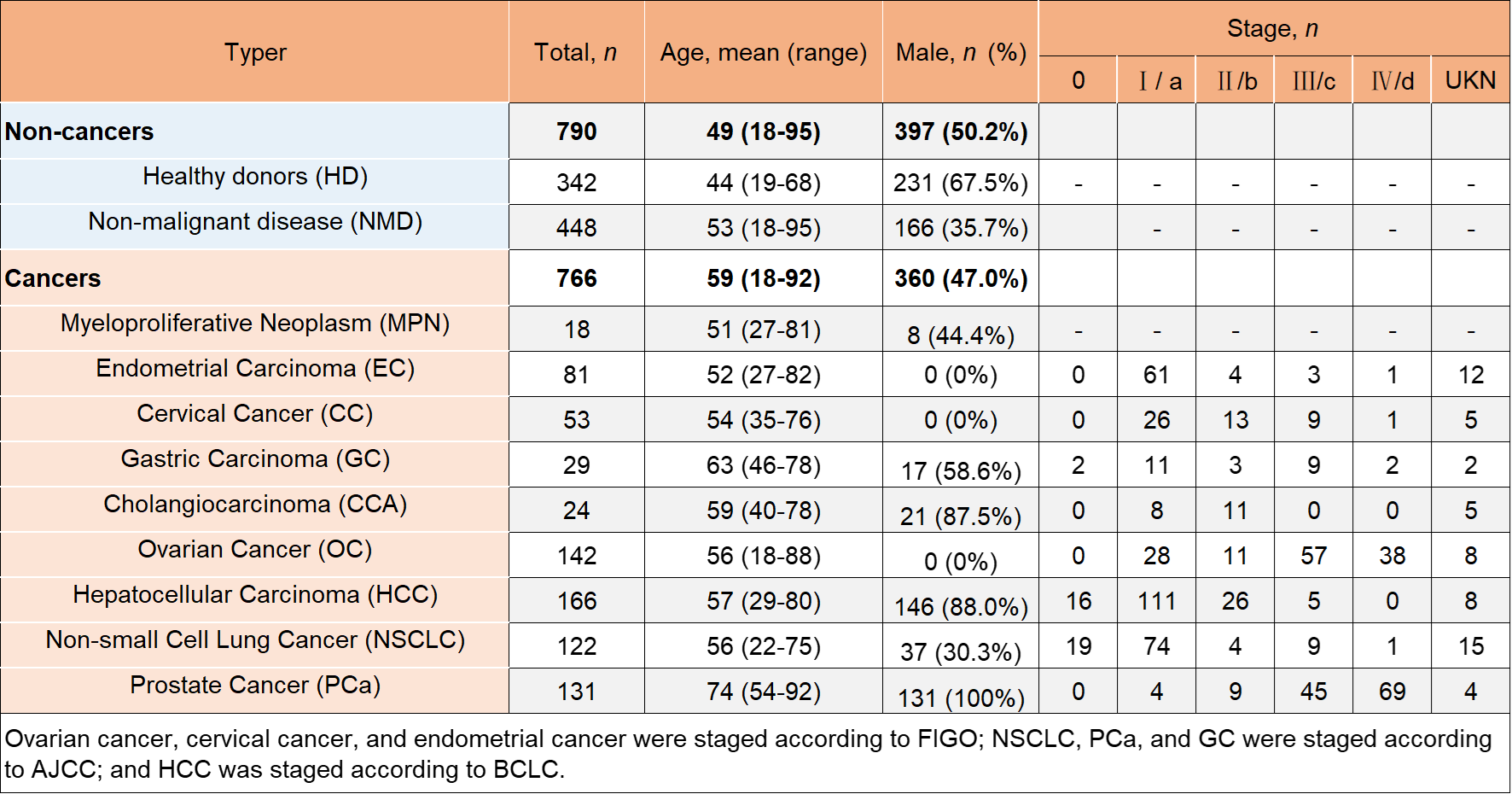


**Extended Data Table 2 | Platelet α-granule distribution patterns in HD, NMD, and patients with nine cancers.**


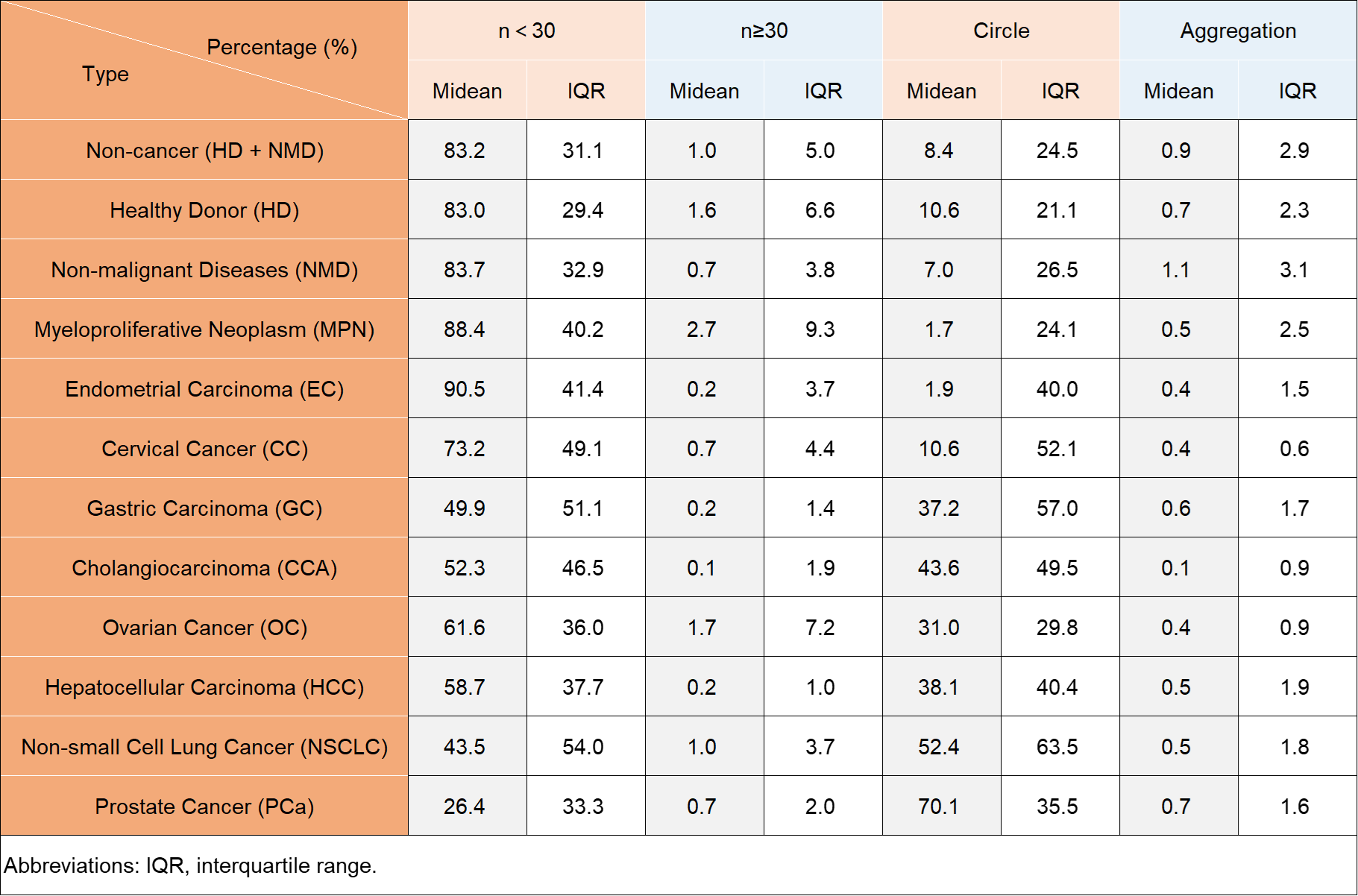


**Extended Data Table 3 | *P* values for differences in platelet α-granule distribution between non-cancer individuals and patients with nine cancers.**


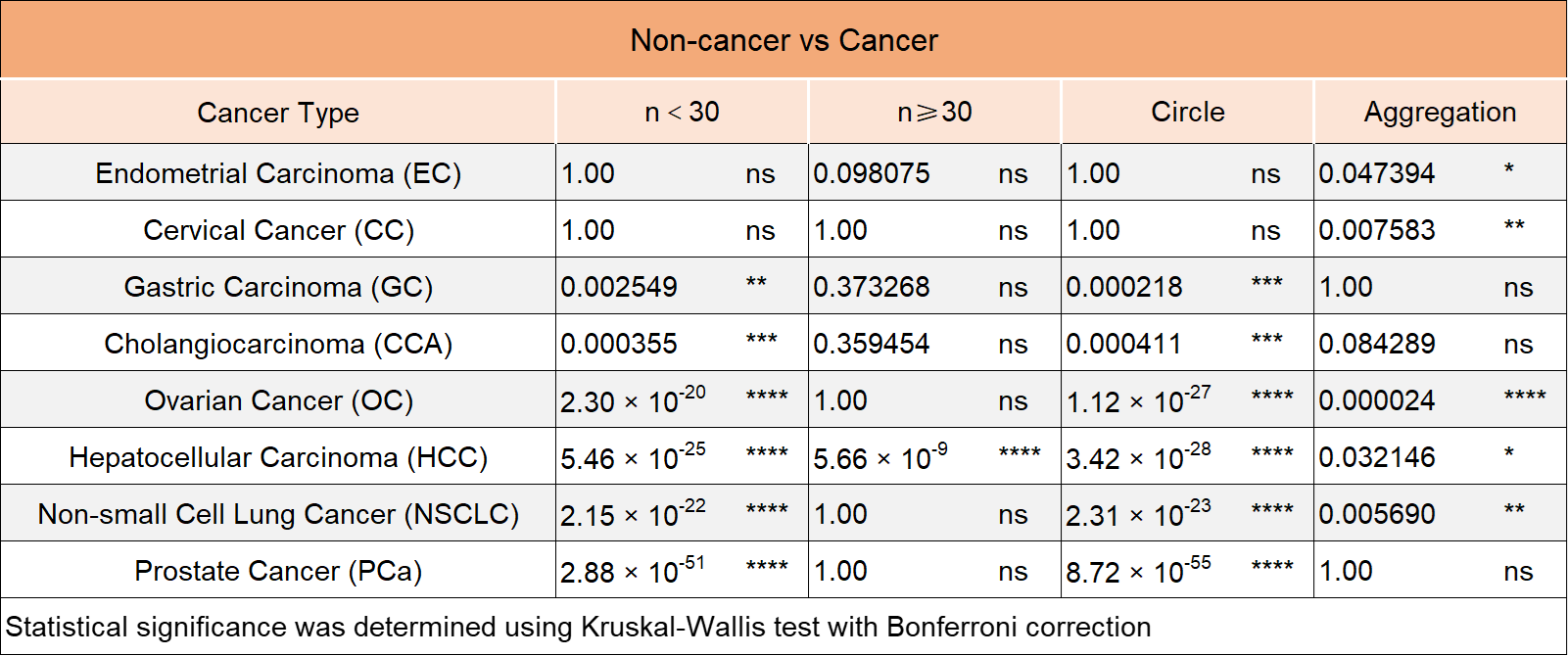


**Extended Data Table 4 |** **ROC analysis results for distinguishing cancer from non-cancer based on PAID.**


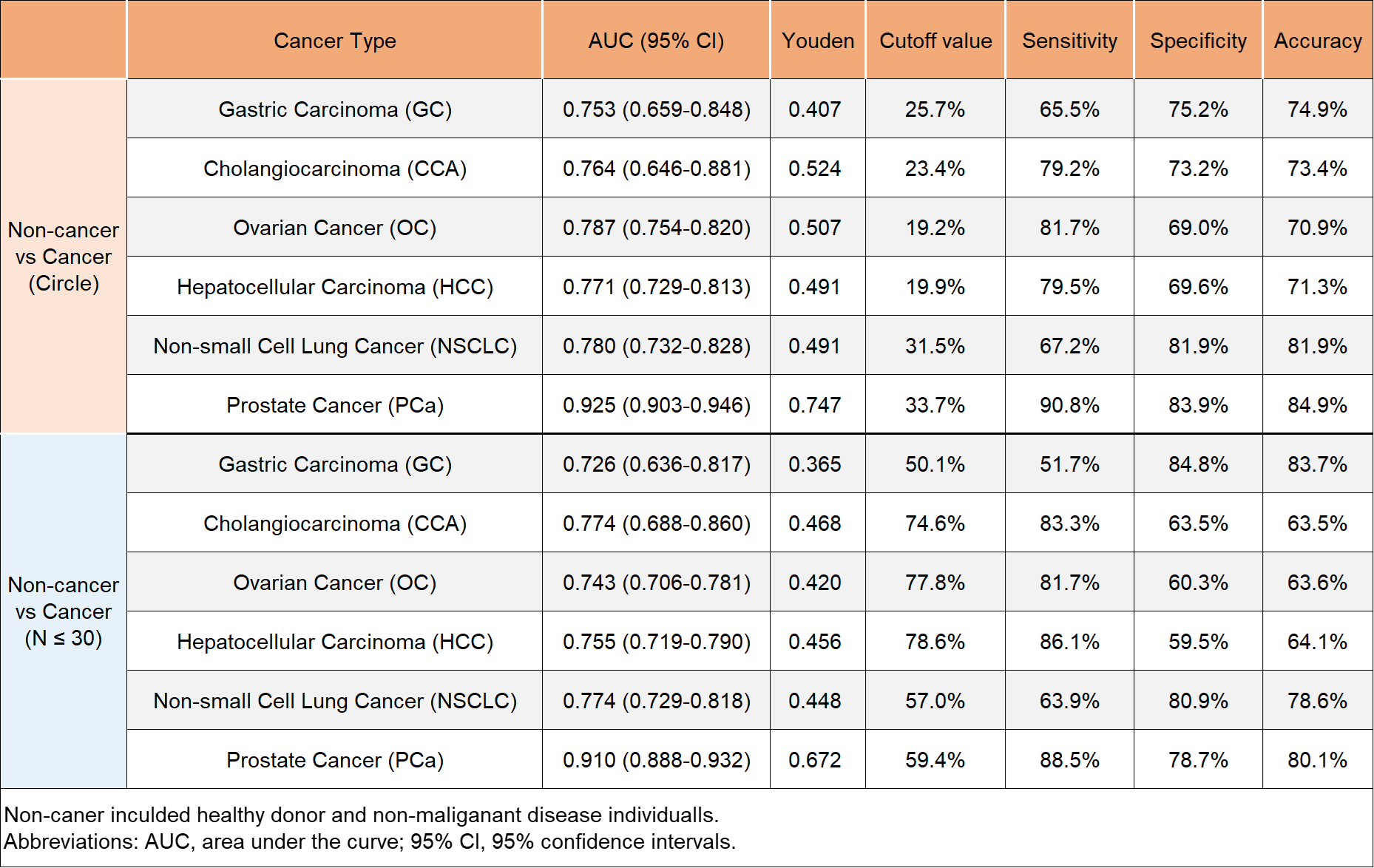


**Extended Data Table 5 | ROC analysis results for distinguishing prostate cancer (PCa) from benign prostatic hyperplasia (BPH) based on PSA, PAID, and their combinations.**

**
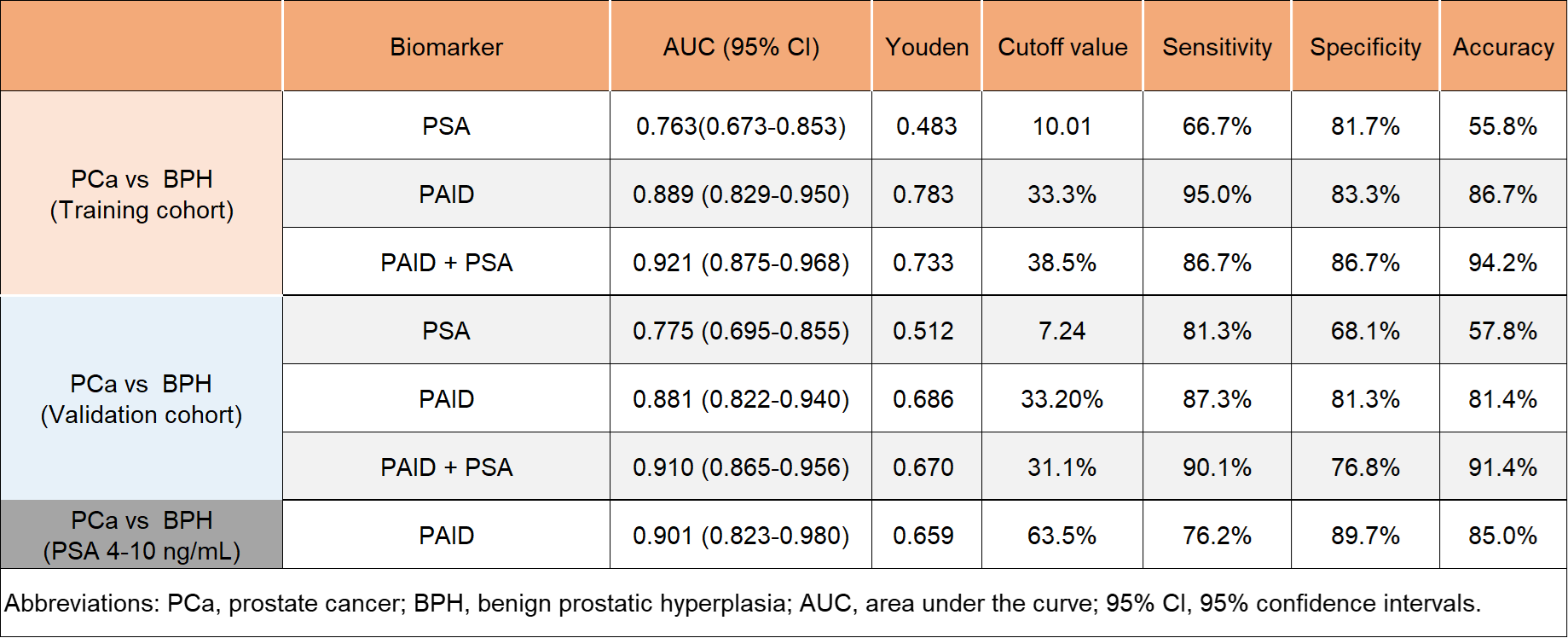
**

**Extended Data Table 6 | ROC analysis results for distinguishing ovarian cancer (OC) from benign adnexal masses (BAM) based on HE4, CA125, PAID, and their combinations.**


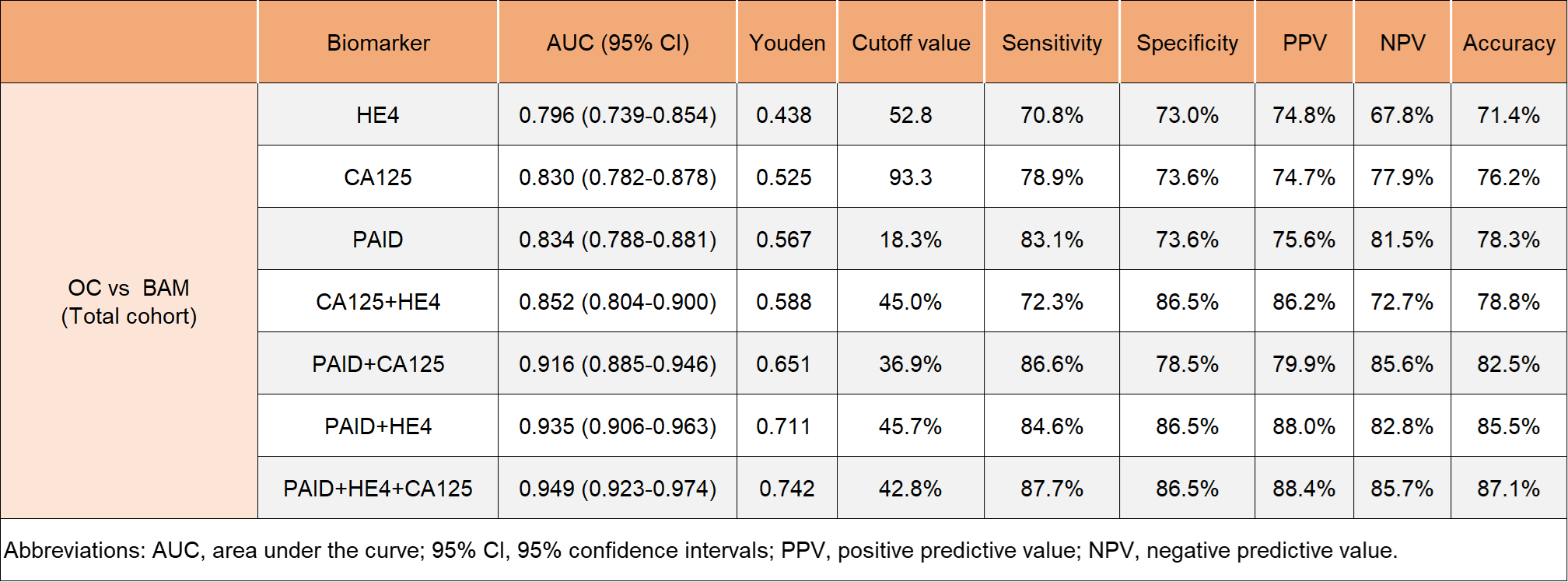


**Extended Data Table 7 | ROC analysis results for detecting OC and recurrent OC based on HE4, CA125, PAID, and their combinations.**


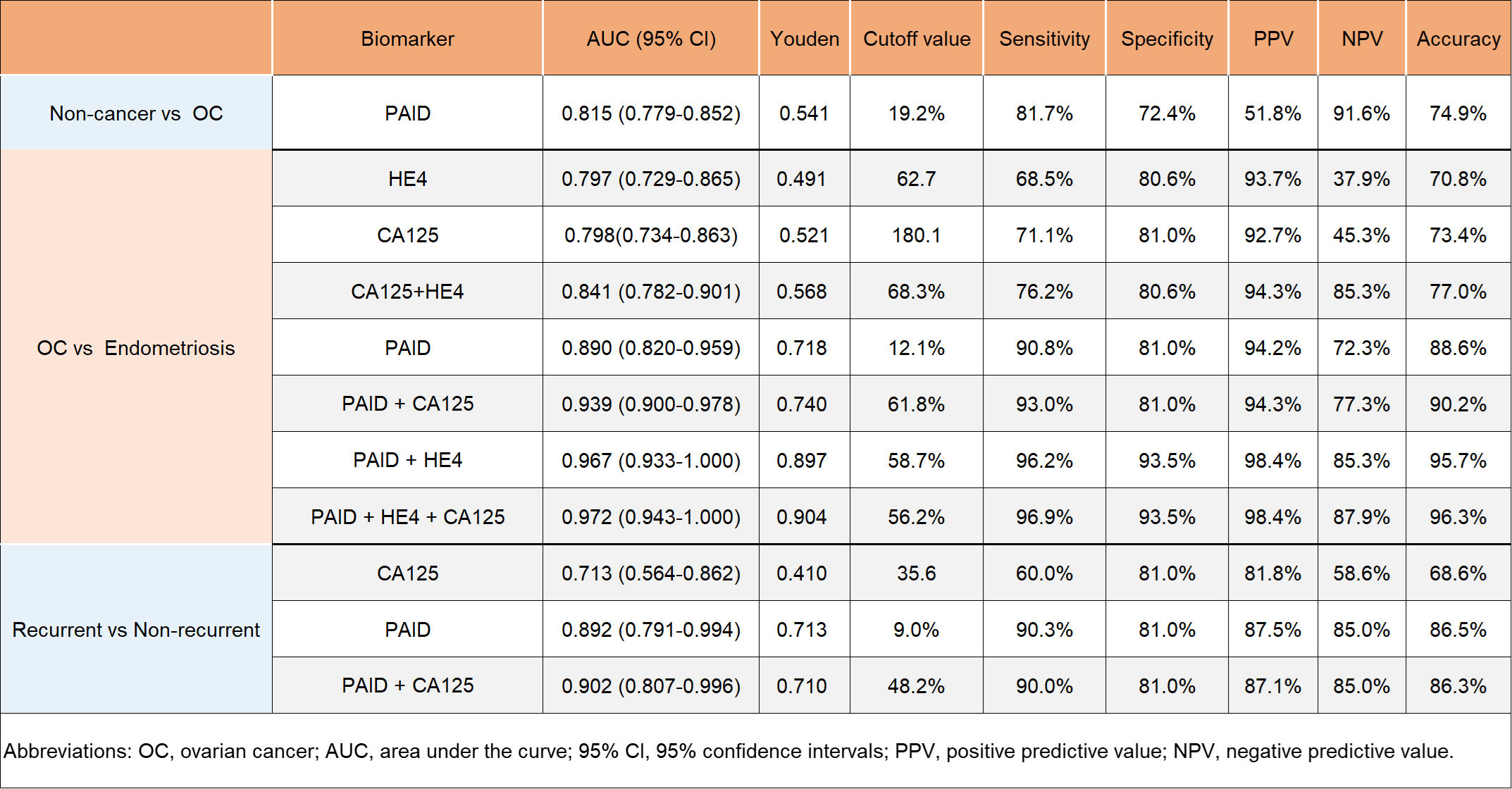


**Extended Data Table 8 | Logistic regression equations for biomarker combinations in the ovarian and prostate cancer diagnostic models.**


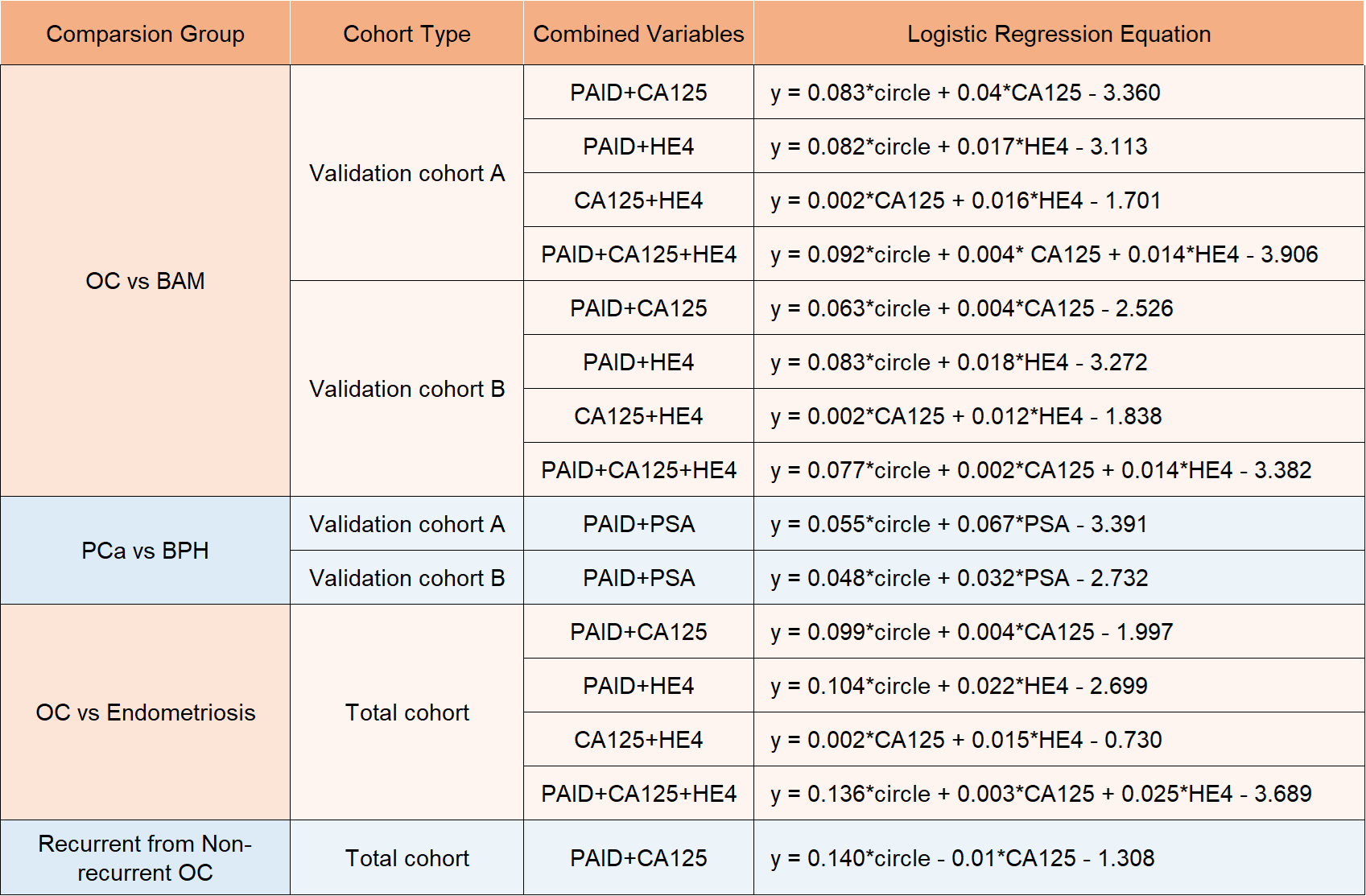


**Section S1. Detailed Description of Model Development and Training**

**S1.1 Platelet Segmentation Model Based on ResUNet**

The model was trained using both fluorescence and bright field images of platelets. The dataset consisted of 60 bright field images, which were manually masked to serve as the gold standard. This dataset was divided into training, validation, and test sets in a ratio of 4:1:1. Prior to training, bright field images were preprocessed by first enlarging their size to 2048×2048 pixels via bilinear interpolation to match the resolution of the fluorescence images, followed by z-score normalization of pixel intensities to correct for illumination differences. The architecture of our ResUNet, detailed in previous report^15^, features down-sampling modules that each contain a 3-pixel convolutional layer with a stride of 1 pixel, a batch normalization layer, a linear activation layer, and a maximum pooling layer to encode high-level semantic information. This network achieved an accuracy of approximately 98.0% on the test set. During the inference stage, the model takes a bright field image as input and returns a segmentation mask. To ensure the analysis was performed on high-quality, single platelets, a series of post-processing steps based on connected component analysis were implemented: (a) removal of adherent or stacked platelets, defined as those with an area greater than 23 μm² or a circularity lower than 0.65; (b) elimination of platelets too close to the image boundary (distance < 0.306 μm) to ensure cell integrity; (c) identification of the central position of each platelet to center it within the image; and (d) cropping of the bright field and corresponding fluorescence image to a 21 μm² area centered on the platelet centroid.

**S1.2 Platelet Subcellular Structure Classification Models Based on ResNet-50**

CNN models based on the ResNet-50 framework was used to classify the distribution patterns of α-granules. The models were trained on a large dataset built from at least 40 samples, comprising approximately 20,000 images of α-granules. This dataset was divided into training, validation, and test sets in a ratio of 3:1:1 The ResNet-50 architecture used is illustrated in our previous work^15^. α-granules were classified into three primary categories: scattered dot, circle, and aggregation. The models demonstrated a strong ability to learn relevant morphological features, as evidenced by the Classification Activation Maps (CAM). Performance was validated by confusion matrices, where the average value of the diagonal elements, representing correct classifications, was approximately 91%^15^. For α-granules initially classified into the 'scattered dot' category, a subsequent quantification step was implemented to further divide them into two subcategories based on the number of granules per platelet (n < 30 or n ≥ 30). This counting was performed automatically by applying a sparse deconvolution algorithm to reduce out-of-focus background and noise, followed by the Otsu method for binary thresholding, achieving a counting accuracy of approximately 95%. During classification, images returned by the segmentation model that had low fluorescence signal or were noisy were automatically filtered out by discarding predictions with a confidence score lower than 0.7.
